# *Echinostoma* '*revolutum*' (Digenea: Echinostomatidae) species complex revisited: species delimitation based on novel molecular and morphological data gathered in Europe

**DOI:** 10.1186/s13071-014-0520-8

**Published:** 2014-11-27

**Authors:** Simona Georgieva, Anna Faltýnková, Rebecca Brown, Isabel Blasco-Costa, Miroslava Soldánová, Jiljí Sitko, Tomáš Scholz, Aneta Kostadinova

**Affiliations:** Institute of Parasitology, Biology Centre of the Academy of Sciences of the Czech Republic, Branišovská 31, 370 05 České Budějovice, Czech Republic; Faculty of Science, University of South Bohemia, Branišovská 31, 370 05 České Budějovice, Czech Republic; College of Medical, Veterinary and Life Sciences, University of Glasgow, Glasgow, G12 8QQ UK; Natural History Museum of Geneva, P.O. Box 6134, CH-1211 Geneva, Switzerland; Komenský Museum, Horní nám, 7, 750 11 Přerov 2, Přerov, Czech Republic

**Keywords:** *Echinostoma* '*revolutum*' species complex, Molecular and morphological data, *nad*1, 28S rDNA, Europe

## Abstract

**Background:**

The systematics of echinostomes within the so-called '*revolutum*' group of the genus *Echinostoma*, which encompasses the type-species *E. revolutum* and a number of morphologically similar species, has long been controversial. Recent molecular studies indicate the existence of more species than previously considered valid, thus stressing the need for wider taxon sampling from natural host populations. This is especially true for Europe where morphological evidence indicates higher species diversity than previously thought, but where molecular data are virtually lacking. This gap in our knowledge was addressed in the present study through an integration of morphological and molecular approaches in the investigation of a dataset with larger taxonomic and geographical coverage.

**Methods:**

More than 20,000 freshwater snails belonging to 16 species were collected during 1998–2012 from various localities in eight countries in Europe. Snail screening provided representative larval isolates for five species of the '*revolutum*' group, identified by their morphology. Adult isolates for four species recovered from natural and experimental infections were also identified. Partial fragments of the mitochondrial *nad*1 and 28S rRNA genes were amplified for 74 and 16 isolates, respectively; these were analysed together with the sequences of *Echinostoma* spp. available on GenBank.

**Results:**

Delineation of the European *Echinostoma* spp. was carried out based on molecular, morphological and ecological data. The large-scale screening revealed infections with five *Echinostoma* spp., including one new species: *E. revolutum* (*sensu stricto*), *E. miyagawai*, *E. paraulum*, *E. bolschewense* and *Echinostoma* n. sp. The newly-generated *nad*1 sequences from Europe fall into six distinct, well-supported, reciprocally monophyletic lineages corresponding to the species identifications based on morphology; this was corroborated by the 28S rDNA sequences. The analyses of the total *nad*1 dataset provided evidence for 12 monophyletic groups and five singletons, which represent seven described/named species and ten cryptic species-level lineages of *Echinostoma*.

**Conclusion:**

We conclude that *nad*1 should be the first choice for large-scale barcode-based identification of the species of the '*revolutum*' group. Our study provides a comprehensive reference library for precisely identified isolates of the European species and highlights the importance of an integrative approach for species identification linking molecular, morphological and biological data.

**Electronic supplementary material:**

The online version of this article (doi:10.1186/s13071-014-0520-8) contains supplementary material, which is available to authorized users.

## Background

The systematics of the echinostomes (Digenea: Echinostomatidae) within the so-called ‘*revolutum*’ group of the genus *Echinostoma* Rudolphi, 1809, which encompasses the type-species *E. revolutum* (Frölich, 1802) and a number of morphologically similar species possessing 37 collar spines, has long been controversial. Problems in defining the species status within this complex include substantial interspecific homogeneity of the morphological characters of both larval and adult stages, inadequate descriptions, poor differential diagnoses and questionable synonymy [1,2] (see Kostadinova & Gibson [3] for a detailed review).

The ‘*revolutum*’ group has been revised twice. Beaver [4] considered only *E. revolutum* valid, and placed nine species (*Distoma echinatum* Zeder, 1803, *Echinostoma miyagawai* Ishii, 1932, *E. cinetorchis* Ando & Ozaki, 1923, *E. armigerum* Barker & Irvine in Barker, 1915, *E. coalitum* Barker & Beaver in Barker, 1915, *E. mendax* Dietz, 1909, *E. paraulum* Dietz, 1909, *E. columbae* Zunker, 1925 and *E. limicoli* Johnson, 1920) in synonymy and listed additional 11 species as “*syn. inq.*”. Kanev and colleagues [5-7] enlarged the ‘*revolutum*’ group to five species, i.e. *E. revolutum* (syns *E. audyi* Lie & Umathevy, 1965, *E. ivaniosi* Mohandas, 1973, *E. paraulum* Dietz, 1909 and *E. revolutum* of Kosupko [8-11]), *E. trivolvis* (Cort, 1914) (syns *E. revolutum* of Beaver [4] and *E. rodriguesi* Hsu, Lie & Basch, 1968), *E. caproni* Richard, 1964 (syns *E. liei* Jeyarasasingam et al., 1972, *E. togoensis* Jourdan & Kulo, 1981 and *E. paraensei* Lie & Basch, 1967), *E. jurini* (Skvortsov, 1924) (syns *E. sisjakowi* Skvortzov, 1934, *E. orlovi* Romashov, 1966 and *E. bolschewense* (Kotova, 1939)) and *E. echinatum* (Zeder, 1803) (syns *Cercaria spinifera* La Valette, 1855, *E. lindoense* Sandground & Bonne, 1940, *E. barbosai* Lie & Basch, 1966, *E. miyagawai* of Kosupko [8-11] and *E. revolutum* of Našincová [12]).

These authors distinguished the five species based mainly on a single morphological feature of their larval stages (the number of outlets of the paraoesophageal gland-cells in the cercaria), the specificity towards the snail first intermediate host (at the familial level), their ability to infect avian or mammalian hosts (or both) and their geographical range on a global scale (continents) (see Kostadinova *et al*. [1] and Kostadinova & Gibson [3] for detailed comments). However, *E. echinatum* cannot be considered valid since this species has not been justified in a taxonomic publication. Further, the re-examination of the voucher specimens from Kanev’s experimental studies used in his delimitation of *E. revolutum* and *E. echinatum* revealed a number of erroneous identifications including members of the genera *Hypoderaeum* Dietz, 1909 and *Echinoparyphium* Dietz, 1909, and a species of *Echinostoma* with 47 collar spines [1,13].

Kanev [5] favoured the idea of allopatric speciation at a continental scale with only two sympatric combinations: (i) *E. revolutum* and *E. echinatum* in Europe and Asia; and (ii) *E. trivolvis* (recorded as its synonym *E. rodriguesi* Hsu, Lie & Basch, 1968), *E. caproni* (recorded as its synonym *E. paraensei* Lie & Basch, 1967) and *E. echinatum* (recorded as its synonym *E. lindoense*) in South America. This simplistic scheme for the ‘*revolutum*’ group has changed since. Based on molecular data, *E. revolutum* was recorded in Australia [14] and North America [15-17], *E. paraensei* was re-validated and recorded in Australia and South America [14,18], and as yet unidentified species/cryptic lineages of the group were distinguished in New Zealand, North America and Europe [14-17,19]. Furthermore, a number of species within the group have been described and/or redescribed based on experimental completion of the life-cycles. These include *E. bolschewense*; *E. friedi* Toledo, Muñoz-Antolí & Esteban, 2000; *E. spiniferum* (La Valette, 1855) *sensu* Našincová [20] and *E. miyagawai* Ishii, 1932 in Europe [1,2,20-22], *E. deserticum* Kechemir, Jourdane & Mas-Coma, 2002 in Africa and *E. luisreyi* Maldonado, Vieira & Lanfredi, 2003 in South America [23,24].

The first molecular study on the problematic ‘*revolutum*’ group found very low levels (1.1–3.7%) of interspecific sequence variation for the nuclear rDNA ITS sequences from isolates of *Echinostoma* spp. maintained in the laboratory [25]. Morgan & Blair [26] obtained sequences of the mitochondrial *cox*1 and *nad*1 genes of these isolates and revealed that the *nad*1 gene provides a better resolution for investigating relationships within this group in comparison with both ITS and *cox*1. These authors used *nad*1 sequences to identify different larval stages of natural echinostome isolates from Australia and New Zealand and reported on the presence of isolates of *E. revolutum* and *E. paraensei* in Australia, plus five additional unidentified species (with more or less than 37 spines), all referred to as “*Echinostoma*” and an unknown species closely related to *E. revolutum* in New Zealand [14]. However, there appeared to be a problem with the identification of the German isolate of *E. revolutum* used by Morgan & Blair [14,25,26] (see Sorensen *et al*. [27] and Kostadinova *et al*. [1,2,28]). Kostadinova *et al*. [28] completed the life-cycle of *E. revolutum* in the laboratory and conducted a molecular study using this Bulgarian isolate and a number of European isolates from species of the genera closely related to *Echinostoma*. These authors provided evidence that the Australian material from Morgan and Blair’s study [14] contained species from different genera (*Isthmiophora* Lühe, 1909, *Hypoderaeum* and *Echinoparyphium*; all referred to as “*Echinostoma*” in GenBank) and that the German and Bulgarian isolates of *E. revolutum* represent different species [3,28].

Recent molecular studies conducted by Detwiler and colleagues in North America suggested the existence of more than ten species of the genera *Echinostoma*, *Echinoparyphium* and *Hypoderaeum* in natural host populations in the USA. These studies confirmed the presence of two species, identified as “*E. revolutum*” and “*E. robustum/friedi*”, and flagged as potentially cryptic taxa divergent lineages for two species, *E. trivolvis* and “*E. robustum/friedi*” the USA [16,17]. Recently, Georgieva *et al*. [19] have shown that the North American isolates of “*E. revolutum*” studied by Detwiler *et al*. [16] represent another cryptic species of the ‘*revolutum*’ species complex and provided molecular and morphological evidence for an as yet undescribed species of *Echinostoma* infecting *Radix* spp. in Germany and Iceland.

In summary, although some of the problems within the ‘*revolutum*’ species complex have been tackled, the results of the recent molecular studies stress the need for (i) a wider taxon sampling from natural host populations, especially in Europe where morphological evidence indicates higher species diversity than previously thought, but where molecular data are virtually lacking, and (ii) an integration of molecular, morphological and biological data and taxonomic expertise as a way forward to achieving high resolution and consistency of the identification of *Echinostoma* spp.

This gap in our knowledge was addressed in the present study through an integration of morphological and molecular approaches in investigation of a dataset with larger taxonomic and geographical coverage. We carried out molecular prospecting (*sensu* Blouin [29]) for the diversity of the European species of *Echinostoma* by generating a sequence database linking *nad*1 and 28S rDNA sequences for larval and adult (experimentally raised and from naturally infected definitive hosts) isolates of *Echinostoma* spp. These were collected in an extensive sampling programme in eight countries in Europe and identified based on parasite morphology. The inclusion of reliably identified species from Europe in the substantially enlarged *nad*1 database and the phylogenetic and distance-based approaches to species delineation applied here further expand the molecular framework for the diversity and distribution of the ‘*revolutum*’ group developed by Morgan & Blair and Detwiler and colleagues that will accelerate the taxonomic revision of this complex of morphologically similar species. Our results considerably enhance the consistency of the identification within this group of cryptic species based on molecular data and thus have implications for both monitoring the diversity and host-parasite relationships of *Echinostoma* spp. and detecting important pathogens in wild host populations and humans.

## Methods

### Sample collection

More than 20,000 freshwater snails belonging to 16 species [*Lymnaea stagnalis* (L.), *Radix auricularia* (L.), *R. peregra* (Müller), *Stagnicola palustris* (Müller), *Planorbis planorbis* (L.), *P. carinatus* Müller, *Planorbarius corneus* (L.), *Anisus leucostoma* (Millet), *A. vortex* (L.), *Bathyomphalus contortus* (L.), *Gyraulus albus* (Müller), *G. acronicus* (Férussac), *G. crista* (L.), *Segmentina nitida* (Müller), *Ancylus fluviatilis* Müller and *Viviparus acerosus* (Bourguignat)] were collected in an extensive sampling programme during 1998–2012 from various localities in eight countries in Europe: Austria, Bulgaria, Czech Republic, Finland, Germany, Hungary, Poland and Slovak Republic. Snails were screened for trematode infections and representative samples of each cercarial isolate (i.e. a group of identical individuals collected from a single host at one point in time [14]) of *Echinostoma* spp. were examined live and fixed in hot 4% formaldehyde solution for obtaining metrical data, and in molecular grade ethanol for DNA isolation (see Table 1 for a list of isolates, their hosts, localities and the accession numbers of the sequences). Cercariae were examined live and identified using the data from the relevant primary sources (e.g. Kosupko [9-11]; Našincová [12,21]; Kostadinova *et al*. [1,2]; Toledo *et al*. [22] and the keys in Faltýnková *et al*. [30,31].

**Table 1:** **Summary data for the isolates of** ***Echinostoma*** **spp. used for generation of the new** ***nad*** **1 and 28S rDNA sequences**

**Species**	**Isolate**	**Life-cycle stage**	**Host species**	**Collection site**	***nad*** **1 haplotype ID**	**GenBank accession number**	
						***nad*** **1**	**28S rDNA**
***E. bolschewense***	EBG1	C	*Viviparus acerosus*	Danube at Gabčíkovo (Slovakia)	1	KP065608	
***E. bolschewense***	EBG2	C	*Viviparus acerosus*	Danube at Gabčíkovo (Slovakia)	1	KP065609	
***E. bolschewense***	EBG3	C	*Viviparus acerosus*	Danube at Gabčíkovo (Slovakia)	1	KP065610	
***E. bolschewense***	EBG4	C	*Viviparus acerosus*	Danube at Gabčíkovo (Slovakia)	1	KP065611	
***E. bolschewense***	EBG5	C	*Viviparus acerosus*	Danube at Gabčíkovo (Slovakia)	1	KP065612	
***E. bolschewense***	EBG6	C	*Viviparus acerosus*	Danube at Gabčíkovo (Slovakia)	1	KP065613	
***E. bolschewense***	EBG7	C	*Viviparus acerosus*	Danube at Gabčíkovo (Slovakia)	1	KP065614	
***E. bolschewense***	EBG8	C	*Viviparus acerosus*	Danube at Gabčíkovo (Slovakia)	1	KP065615	
***E. bolschewense***	EBG9	C	*Viviparus acerosus*	Danube at Gabčíkovo (Slovakia)	1	KP065616	
***E. bolschewense***	EBG10	C	*Viviparus acerosus*	Danube at Gabčíkovo (Slovakia)	1	KP065617	
***E. bolschewense***	EBG11	C	*Viviparus acerosus*	Danube at Gabčíkovo (Slovakia)	1	KP065618	
***E. bolschewense***	EBG12	C	*Viviparus acerosus*	Danube at Gabčíkovo (Slovakia)	1	KP065619	
***E. bolschewense***	EBG13	C	*Viviparus acerosus*	Danube at Gabčíkovo (Slovakia)	1	KP065620	KP065591
***E. bolschewense***	EBG14	C	*Viviparus acerosus*	Danube at Gabčíkovo (Slovakia)	2	KP065621	KP065592
***E. bolschewense***	EBG15	C	*Viviparus acerosus*	Danube at Gabčíkovo (Slovakia)	2	KP065622	
***E. bolschewense***	EBG16	C	*Viviparus acerosus*	Danube at Gabčíkovo (Slovakia)	2	KP065623	
***E. miyagawai***	EMGD1	A	*Anas platyrhynchos*	Vicinities of Gdańsk (Poland)	1	KP065624	
***E. miyagawai***	EMT1	A	*Aythya fuligula*	Vicinities of Tovačov (Czech Republic)	1	KP065625	
***E. miyagawai***	EML1	C	*Planorbis planorbis*	Pond Loužek (Czech Republic)	2	KP065626	
***E. miyagawai***	EML2	C	*Planorbis planorbis*	Pond Loužek (Czech Republic)	2	KP065627	
***E. miyagawai***	EML3	C	*Planorbis planorbis*	Pond Loužek (Czech Republic)	3	KP065628	
***E. miyagawai***	EML4	C	*Planorbis planorbis*	Pond Loužek (Czech Republic)	3	KP065629	
***E. miyagawai***	EML5	C	*Planorbis planorbis*	Pond Loužek (Czech Republic)	4	KP065630	
***E. miyagawai***	EML6	C	*Planorbis planorbis*	Pond Loužek (Czech Republic)	4	KP065631	
***E. miyagawai***	EML7	C	*Planorbis planorbis*	Pond Loužek (Czech Republic)	5	KP065632	
***E. miyagawai***	EML8	C	*Planorbis planorbis*	Pond Loužek (Czech Republic)	6	KP065633	
***E. miyagawai***	EML9	C	*Planorbis planorbis*	Pond Loužek (Czech Republic)	7	KP065634	
***E. miyagawai***	EML10	C	*Planorbis planorbis*	Pond Loužek (Czech Republic)	8	KP065635	
***E. miyagawai***	EML11	C	*Planorbis planorbis*	Pond Loužek (Czech Republic)	9	KP065636	
***E. miyagawai***	EML12	C	*Planorbis planorbis*	Pond Loužek (Czech Republic)	10	KP065637	
***E. miyagawai***	EML13	C	*Planorbis planorbis*	Pond Loužek (Czech Republic)	11	KP065638	
***E. miyagawai***	EMGD2	A	*Anas platyrhynchos*	Vicinities of Gdańsk (Poland)	12	KP065639	
***E. miyagawai***	EMT2	A	*Aythya fuligula*	Vicinities of Tovačov (Czech Republic)	13	KP065640	KP065593
***E. miyagawai***	EML14	C	*Planorbis planorbis*	Pond Loužek (Czech Republic)	14	KP065641	
***E. revolutum*** **(** ***s. str.*** **)**	ERBO1	C	*Lymnaea stagnalis*	Lake Bodensee (Germany)	1	KP065642	
***E. revolutum*** **(** ***s. str.*** **)**	ERBA1	C	*Lymnaea stagnalis*	Pond Bartoňovský (Czech Republic)	1	KP065643	KP065594
***E. revolutum*** **(** ***s. str.*** **)**	ERVD1	C	*Lymnaea stagnalis*	Pond Velký Dvorecký (Czech Republic)	1	KP065644	KP065595
***E. revolutum*** **(** ***s. str.*** **)**	ERHH1	C	*Lymnaea stagnalis*	Pond Hluboký u Hamru (Czech Republic)	1	KP065645	
***E. revolutum*** **(** ***s. str.*** **)**	ERV1	C	*Lymnaea stagnalis*	Pond Vlkovský (Czech Republic)	1	KP065646	
***E. revolutum*** **(** ***s. str.*** **)**	ERV2	C	*Lymnaea stagnalis*	Pond Vlkovský (Czech Republic)	1	KP065647	
***E. revolutum*** **(** ***s. str.*** **)**	ERPL1	C	*Radix auricularia*	Pond near Tomislawice (Poland)	1	KP065648	
***E. revolutum*** **(** ***s. str.*** **)**	ERBAL1	C	*Lymnaea stagnalis*	Lake Baldeneysee (Germany)	2	KP065649	
***E. revolutum*** **(** ***s. str.*** **)**	ERV3	C	*Lymnaea stagnalis*	Pond Vlkovský (Czech Republic)	3	KP065650	
***E. revolutum*** **(** ***s. str.*** **)**	ERBAL2	C	*Lymnaea stagnalis*	Lake Baldeneysee (Germany)	4	KP065651	
***E. revolutum*** **(** ***s. str.*** **)**	ERH1	C	*Lymnaea stagnalis*	Lake Hengsteysee (Germany)	5	KP065652	
***E. revolutum*** **(** ***s. str.*** **)**	ERT1	A	*Aythya fuligula*	Vicinities of Tovačov (Czech Republic)	6	KP065653	KP065596
***E. revolutum*** **(** ***s. str.*** **)**	ERHU1	C	*Lymnaea stagnalis*	Lake Huumojärvi, Oulu (Finland)	7	KP065654	
***E. revolutum*** **(** ***s. str.*** **)**	ERHU2	C	*Lymnaea stagnalis*	Lake Huumojärvi, Oulu (Finland)	8	KP065655	
***E. revolutum*** **(** ***s. str.*** **)**	ERK1	C	*Lymnaea stagnalis*	Pond near Krausenbechhofen (Germany)	9	KP065656	
***E. revolutum*** **(** ***s. str.*** **)**	ERHH2	C	*Lymnaea stagnalis*	Pond Hluboký u Hamru (Czech Republic)	10	KP065657	KP065597
***E. revolutum*** **(** ***s. str.*** **)**	ERHH3	C	*Lymnaea stagnalis*	Pond Hluboký u Hamru (Czech Republic)	11	KP065658	KP065598
***E. revolutum*** **(** ***s. str.*** **)**	ERHH4	C	*Stagnicola palustris*	Pond Hluboký u Hamru (Czech Republic)	–	–	KP065599
***Echinostoma*** **n. sp.**	ENG1	C	*Planorbarius corneus*	Danube at Gabčíkovo (Slovakia)	1	KP065659	
***Echinostoma*** **n. sp.**	ENG2	C	*Planorbarius corneus*	Danube at Gabčíkovo (Slovakia)	1	KP065660	
***Echinostoma*** **n. sp.**	ENG3	C	*Planorbarius corneus*	Danube at Gabčíkovo (Slovakia)	1	KP065661	
***Echinostoma*** **n. sp.**	ENB1	C	*Planorbarius corneus*	Pond Bohdaneč (Czech Republic)	1	KP065662	
***Echinostoma*** **n. sp.**	ENV1	C	*Planorbarius corneus*	Pond Vlkovský (Czech Republic)	1	KP065663	
***Echinostoma*** **n. sp.**	ENB2	C	*Planorbarius corneus*	Pond Bohdaneč (Czech Republic)	2	KP065664	
***Echinostoma*** **n. sp.**	ENB3	C	*Planorbarius corneus*	Pond Bohdaneč (Czech Republic)	2	KP065665	
***Echinostoma*** **n. sp.**	ENHH1	C	*Planorbarius corneus*	Pond Hluboký u Hamru (Czech Republic)	3	KP065666	
***Echinostoma*** **n. sp.**	ENV2	C	*Planorbarius corneus*	Pond Vlkovský (Czech Republic)	3	KP065667	KP065600
***Echinostoma*** **n. sp.**	ENHH2	C	*Planorbarius corneus*	Pond Hluboký u Hamru (Czech Republic)	4	KP065668	
***Echinostoma*** **n. sp.**	ENV3	C	*Planorbarius corneus*	Pond Vlkovský (Czech Republic)	4	KP065669	
***Echinostoma*** **n. sp.**	ENG4	C	*Planorbarius corneus*	Danube at Gabčíkovo (Slovakia)	5	KP065670	
***Echinostoma*** **n. sp.**	ENG5	C	*Planorbarius corneus*	Danube at Gabčíkovo (Slovakia)	6	KP065671	
***Echinostoma*** **n. sp.**	ENG6	C	*Planorbarius corneus*	Danube at Gabčíkovo (Slovakia)	7	KP065672	
***Echinostoma*** **n. sp.**	ENV4	C	*Planorbarius corneus*	Pond Vlkovský (Czech Republic)	8	KP065673	
***Echinostoma*** **n. sp.**	ENHH3	C	*Planorbarius corneus*	Pond Hluboký u Hamru (Czech Republic)	9	KP065674	KP065601
***Echinostoma*** **n. sp.**	ENV5	C	*Planorbarius corneus*	Pond Vlkovský (Czech Republic)	10	KP065675	
***Echinostoma*** **n. sp.**	ENBOH1	C	*Planorbarius corneus*	Pond Bohumilečský (Czech Republic)	11	KP065676	
***Echinostoma*** **n. sp.**	ENB4	C	*Planorbarius corneus*	Pond Bohdaneč (Czech Republic)	–	–	KP065602
***Echinostoma*** **n. sp.**	ENV6	C	*Planorbarius corneus*	Pond Vlkovský (Czech Republic)	–	–	KP065603
***E. paraulum***	EPP1	C	*Lymnaea stagnalis*	Pond near Poppenwind (Germany)	1	KP065677	
***E. paraulum***	EPP2	C	*Lymnaea stagnalis*	Pond near Poppenwind (Germany)	1	KP065678	
***E. paraulum***	EPM1	C	*Lymnaea stagnalis*	Nature Reserve Mohrhof (Germany)	2	KP065679	KP065604
***E. paraulum***	EPT1	A	*Aythya fuligula*	Vicinities of Tovačov (Czech Republic)	3	KP065680	KP065605
***E. paraulum***	EPM2	C	*Lymnaea stagnalis*	Nature Reserve Mohrhof (Germany)	4	KP065681	
***Echinostoma*** **sp. IG**	EIGH	C	*Radix auricularia*	Lake Hengsteysee (Germany)	2	KC618449*	KP065606
***Hypoderaeum conoideum***	AK44	C	*Lymnaea stagnalis*	Pond Bartoňovský (Czech Republic)	–	–	KP065607

*Published by Georgieva *et al*. [19].

Experimental completion of the life-cycle was carried out for two species (*E. revolutum* sampled in Bulgaria and *E. paraulum* sampled in Germany) and adult worms were available for morphological identification from the experiments of Našincová [12,20,21] for *E. bolschewense* and *Echinostoma* n. sp. Sequences were also generated from adult isolates of *E. revolutum*, *E. miyagawai* and *E. paraulum* recovered from bird definitive hosts in the wild: *Anas platyrhynchos* (L.) and *Aythya fuligula* (L.) collected in Poland (vicinities of Gdańsk) and the Czech Republic (vicinities of Tovačov), respectively (see Table 1 for details). All adults were identified prior to sequencing on morphological grounds following Kostadinova *et al*. [1,2,28].

### Sequence generation

Total genomic DNA was isolated from alcohol-fixed isolates of cercariae or adult worms (posterior fifth of body, the remainder of the worm kept as voucher) using the protocols of Tkach & Pawlowski [32] or Georgieva *et al*. [19]. Polymerase chain reaction (PCR) amplifications were performed in 25 μl reactions using illustra puReTaq Ready-To-Go PCR Beads (GE Healthcare, UK) containing ~2.5 units of puReTaq DNA polymerase, 10 mM Tris–HCl (pH 9.0), 50 mM KCl, 1.5 mM MgCl_2_, 200 μM of each dNTP and stabilisers including BSA, 10 pmol of each PCR primer, and 50 ng of genomic DNA.

Partial fragments of the mitochondrial gene nicotinamide adenine dinucleotide dehydrogenase subunit 1 (*nad*1) gene were amplified using the primers NDJ11 (forward; 5'-AGA TTC GTA AGG GGC CTA ATA-3' [26]) and NDJ2A (reverse; 5'-CTT CAGCCT CAG CAT AAT-3' [28]). The PCR thermocycling profile comprised initial denaturation at 95°C for 5 min, followed by 35 cycles (30 s denaturation at 94°C, 20 s primer annealing at 48°C, and 45 s at 72°C for primer extension), with a final extension step of 4 min at 72°C. Partial (domains D1–D3; c. 1,400 nt) 28S rDNA sequences were amplified using primer combinations U178F (5'-GCA CCC GCT GAA YTT AAG-3') and L1642R (5'-CCA GCG CCA TCC ATT TTC A-3') [33] or ZX-1 (5'-ACC CGC TGA ATT TAA GCA TAT-3') [34] and 1500R (5'-GCT ATC CTG AGG GAA ACT TCG-3') [35] with the following PCR profile: initial denaturation at 95°C for 5 min, followed by 40 cycles (30 s denaturation at 95°C, 30 s primer annealing at 55°C, and 45 s at 72°C for primer extension), and a final extension step of 7 min at 72°C.

PCR amplicons were purified using either a QIAquick™ Gel Extraction Kit or a Qiagen QIAquick™ PCR Purification Kit (Qiagen Ltd., UK) and sequenced directly for both strands using the PCR primers [plus LSU1200R (5'-CAT AGT TCA CCA TCT TTC GG-3' [33]) for 28S rDNA]. Sequencing was performed on an ABI Prism 3130xl automated sequencer using ABI Big Dye chemistry (ABI Perkin-Elmer, UK) according to the manufacturer’s protocol. Contiguous sequences were assembled and edited using MEGA v6 [36] and submitted to GenBank (accession numbers shown in Table 1).

### Alignments and data analysis

Newly-generated and published *nad*1 and 28S rDNA sequences for *Echinostoma* spp. (Table 1; Additional file 1: Table S1) were aligned using Muscle implemented in MEGA v6; *nad*1 dataset was aligned with reference to the amino acid translation, using the echinoderm and flatworm mitochondrial code [37], but analysed solely as nucleotides (first, second and third positions within the included codons were included in the analyses). Species boundaries were inferred with the application of the Neighbour-Joining (NJ) method using the Kimura’s 2 parameter model (K2P) of substitution for pairwise distance calculations with MEGA v6 (1,000 bootstrap replicates) and Bayesian inference (BI) analyses using MrBayes v3.2 [38]. The best-fitting models of nucleotide substitution were estimated prior to BI analyses with jModelTest 2.1.4 [39,40]. These were the general time reversible model, with estimates of invariant sites and gamma distributed among-site rate variation (GTR + I + G) (*nad*1 dataset) and Hasegawa-Kishino-Yano model including estimates of invariant sites (HKY + I) (28S rDNA dataset). Log-likelihoods were estimated over 10^6^ generations *via* 4 simultaneous Markov Chain Monte Carlo chains (nchains = 4) with a sampling frequency of 100. The first 25% of the samples were discarded (sump burnin = 2,500) as determined by the stationarity of lnL assessed with Tracer v.1.4 [41]; the remaining trees were used to construct the 50% majority-rule consensus tree and to estimate the nodal support as posterior probability values [42]. Genetic distances (uncorrected p-distance) were calculated with MEGA v6. Non-metric multidimensional scaling (NMDS) ordination performed with Primer v6 software [43] was used to visualise the raw pairwise distances. The significance of the relationship between the mean intra-specific divergence and the number of isolates sequenced was assessed with Spearman’s correlation.

In addition to tree-based approaches to species delineation we used the distance-based identification method implemented in the function Species Identifier v1 within the program TAXONDNA [44]. The algorithm performs assignment to the correct species using K2P pairwise distances in comparisons of each sequence against the dataset using the “best close match” criterion. Assignment outcome is considered successful if the sequences exhibiting the lowest genetic distance (closest matches) are conspecific with the query sequence and the distance between the query and closest matches falls below a specified threshold. We used a distance threshold of 3%, which is a more conservative estimate than the two threshold values calculated after Meier *et al*. [44], i.e. 0.84% (distance below which 95% of all pairwise comparisons are found; n = 825) and 2.74% (distance below which 99% of all pairwise comparisons are found; n = 1,631). Relationships between haplotypes of *E. revolutum sensu lato* (*s.l*.) from Europe and North America were visualised with haplotype networks constructed with statistical parsimony analysis using TCS version 1.21 [45].

### Species delineation

Delineation of the European species of *Echinostoma* was based on the integration of molecular, morphological and ecological data: (i) support for reciprocal monophyly in the *nad*1 phylogeny (a conservative approach to species delimitation); (ii) pairwise divergence at *nad*1 (including distance-based assignment) and 28S rRNA genes; (iii) matching of sequences for larval and adult stages (three of the species); (iv) comparisons with already published sequences; (v) morphological characterisation and identification of the cercarial and adult isolates; (vi) inference from the experimental completion of life-cycles (all five species); (vi) the use of different first intermediate hosts.

## Results

### Infections in natural host populations

The large-scale screening of natural snail populations in Europe revealed infections with five *Echinostoma* spp., including one species new to science: *E. revolutum* (type-species), *E. miyagawai*, *E. paraulum*, *E. bolschewense* and *Echinostoma* n. sp. Considering the recent results of Georgieva *et al*. [19] who delineated another putative new species (*Echinostoma* sp. IG), eight snail species are found to be infected with *Echinostoma* spp. in Europe, namely the lymnaeids *Lymnaea stagnalis*, *Radix auricularia*, *R. peregra* and *Stagnicola palustris*; the planorbids *Planorbis planorbis, Anisus vortex* and *Planorbarius corneus*; and the viviparid *Viviparus acerosus*. Five species acted as hosts of a single species of *Echinostoma*: *A. vortex* (*E. miyagawai*), *S. palustris* (*E. revolutum*), *P. planorbis* (*E. miyagawai*), *P. corneus* (*Echinostoma* n. sp.) and *V. acerosus* (*E. bolschewense*) and three lymnaeids hosted two *Echinostoma* spp. each: *L. stagnalis* (*E. revolutum* and *E. paraulum*), *R. auricularia* and *R. peregra* (*E. revolutum* and *Echinostoma* sp. IG) (see also [19]). *Echinostoma revolutum* exhibited the widest host range being recovered in the four lymnaeids studied (*L. stagnalis*, *R. auricularia*, *R. peregra* and *S. palustris*).

All cercariae exhibited characteristic features of the species belonging to the ‘*revolutum*’ species complex of *Echinostoma*: (i) 37 collar spines with an arrangement 5-6-15-6-5 (5 angle and 6 lateral spines on each side and 15 dorsal spines in a double row); (ii) tail with a tip forming a highly contractile attenuated process and seven prominent tegumental fin-folds (2 dorsal, 3 ventral and 2 ventrolateral); and (iii) a flame-cell formula 2[(3 + 3 + 3) + (3 + 3 + 3)] = 36 [19]. However, detailed examination of cercarial morphology revealed specific differences with respect to a combination of characters, i.e. the number and distribution of the penetration and para-oesophageal gland-cells and the structure of the tail fin-folds (see Faltýnková *et al*. [46]).

Adult isolates representing four species were identified, three (*E. revolutum*, *E. miyagawai* and *E. paraulum*) recovered from naturally infected *Aythya fuligula* and *Anas platyrhynchos* and experimentally-raised specimens of *E. revolutum* and *E. paraulum*. In both life-cycle experiments the *nad*1 sequences of the adults were identical with the sequences of the cercariae used as starting material for infection (see also [28]). Morphological descriptions and sequences for *Echinostoma* sp. IG based on cercarial isolates sampled in Germany and Iceland have been published recently (Georgieva *et al*. [19]; see also Additional file 1: Table S1 for details). Formal description of this putative new species awaits the discovery of the adult stage. Detailed descriptions of the life-cycle stages of *Echinostoma* spp. from Europe and formal naming of the new species reported here will be published elsewhere [46], in order to avoid nomenclatural problems due to uncertainty concerning the first publication of the name.

### Novel molecular data from Europe

Our study generated 74 novel partial *nad*1 sequences for five of the six European species of *Echinostoma* included in the analyses; these were collapsed into 39 unique haplotypes. Considering the sequences generated by Kostadinova *et al*. [28] and Georgieva *et al*. [19], the European *nad*1 dataset for *Echinostoma* spp. represented a total of 88 sequences and 50 unique haplotypes. Twenty haplotypes were identified in isolates of *E. revolutum* from four snail host species [*L. stagnalis* (ten haplotypes), *R. auricularia* (four haplotypes), *R. peregra* (seven haplotypes) and *S. palustris* (one haplotype)] with wide distribution in Germany (five localities), Czech Republic (four localities), Poland, Iceland, Finland and Bulgaria (one locality each) (Table 1; Additional file 1: Table S1). There was no differentiation within Europe (Table 2) with identical haplotypes shared across localities separated by as much as 2,500 km (haplotype 1, the most abundant haplotype found in *L. stagnalis* and *Radix* spp; see Table 1 and Additional file 1: Table S1).

**Table 2:** **Mean percent intraspecific (along the diagonal) and interspecific divergence (below the diagonal) for** ***Echinostoma*** **spp. in the** ***nad*** **1 dataset and number of pairwise nucleotide differences for 28S rDNA sequences (above the diagonal)**

		**1**	**2**	**3**	**4**	**5**	**6**	**7**	**8**	**9**	**10**	**11**	**12**	**13**	**14**	**15**
**1**	***E. bolschewense***	**0.07**	12–13	11–12	18	11–12	13–16	12	8	–	–	–	13	–	–	–
**2**	***Echinostoma*** **n. sp.**	16.5	**0.53**	3–4	10	3–4	13	4	7	–	–	–	5	–	–	–
**3**	***E. miyagawai***	16.5	14.0	**0.83**	9	3–4	10	13	11	–	–	–	7	–	–	–
**4**	***E. revolutum*** **(** ***s. str.*** **) (Europe)**	14.3	13.0	11.4	**0.83**	9–10	15	12	13	–	–	–	13	–	–	–
**5**	***E. paraulum***	15.8	15.1	10.8	12.6	**0.55**	13–16	5–6	6–7	–	–	–	6–7	–	–	–
**6**	***Echinostoma*** **sp. IG**	19.3	18.9	19.0	18.2	19.4	**0.32**	13	11	–	–	–	16	–	–	–
**7**	***E. paraensei***	17.0	12.6	15.9	14.9	15.3	19.3	**0.21**	8	–	–	–	5	–	–	–
**8**	***E. caproni***	18.0	15.3	14.4	15.0	14.8	19.3	14.6	**1.82**	–	–	–	9	–	–	–
**9**	**"** ***E. robustum/friedi*** **" Lineage A**	16.9	13.9	4.9	11.3	10.8	17.3	15.3	14.0	**–**	–	–	–	–	–	–
**10**	**"** ***E. robustum/friedi*** **" Lineage C**	15.7	13.3	9.2	10.9	10.2	18.9	13.6	14.2	8.4	**–**	–	–	–	–	–
**11**	**"** ***E. robustum/friedi*** **" Lineage D**	16.9	13.1	8.4	12.2	10.6	19.1	14.7	15.4	8.6	5.3	**–**	–	–	–	–
**12**	***E. trivolvis*** Lineage A*	16.3	11.8	14.6	13.0	14.7	18.0	13.6	14.3	14.1	12.7	12.9	**0.80**	–	–	–
**13**	***E. trivolvis*** **Lineage B**	15.6	12.7	15.2	14.0	15.8	19.6	14.3	16.6	14.9	14.0	13.0	8.1	**0.91**	–	–
**14**	***E. trivolvis*** **Lineage C**	14.4	11.2	15.5	13.5	15.8	19.0	14.1	16.6	15.6	13.2	13.0	7.9	2.7	**0.46**	–
**15**	**"** ***E. revolutum*** **" (USA)**	15.2	13.2	12.0	5.9	13.3	18.8	15.6	14.4	11.8	11.7	13.5	13.9	14.6	13.6	**0.88**

*28S rDNA sequence (AY222246) published as *E. revolutum* by Olson *et al*. [47].

Although most of the isolates of *E. miyagawai* originated from a single locality in the Czech Republic, we found high haplotype diversity (14 haplotypes). Notably, one haplotype was shared between adult isolates ex *An. platyrhynchos* from Poland and *Ay. fuligula* from the Czech Republic, “*E. revolutum* Germany, Europe” (AF025832) of Morgan & Blair [14,26] and *E. friedi* (Valencia, Spain; AJ564379), i.e. across localities separated by as much as 2,200 km. In contrast, *E. bolschewense*, a species that was also sampled at a single locality, was represented by two haplotypes; the most common haplotype (n = 13) was found at three closely-located sites within two different years.

Eleven haplotypes were identified from isolates of *Echinostoma* n. sp.; the most common haplotype was shared between locations in Slovakia (Gabčíkovo) and both northern (Pond Bohdaneč) and southern (Pond Vlkovský) locations in the Czech Republic. The two under-sampled (presumably rare) species, *Echinostoma* sp. IG and *E. paraulum*, were represented by three and four haplotypes, respectively. One haplotype of *Echinostoma* sp. IG was shared between cercarial isolates from *R. peregra* in Iceland and Wales, UK (AY168937), the latter provisionally identified on the basis of cercarial morphology as *E*. cf. *friedi* by Kostadinova *et al*. [28].

### Phylogeny-based species delimitation

**Figure 1:**
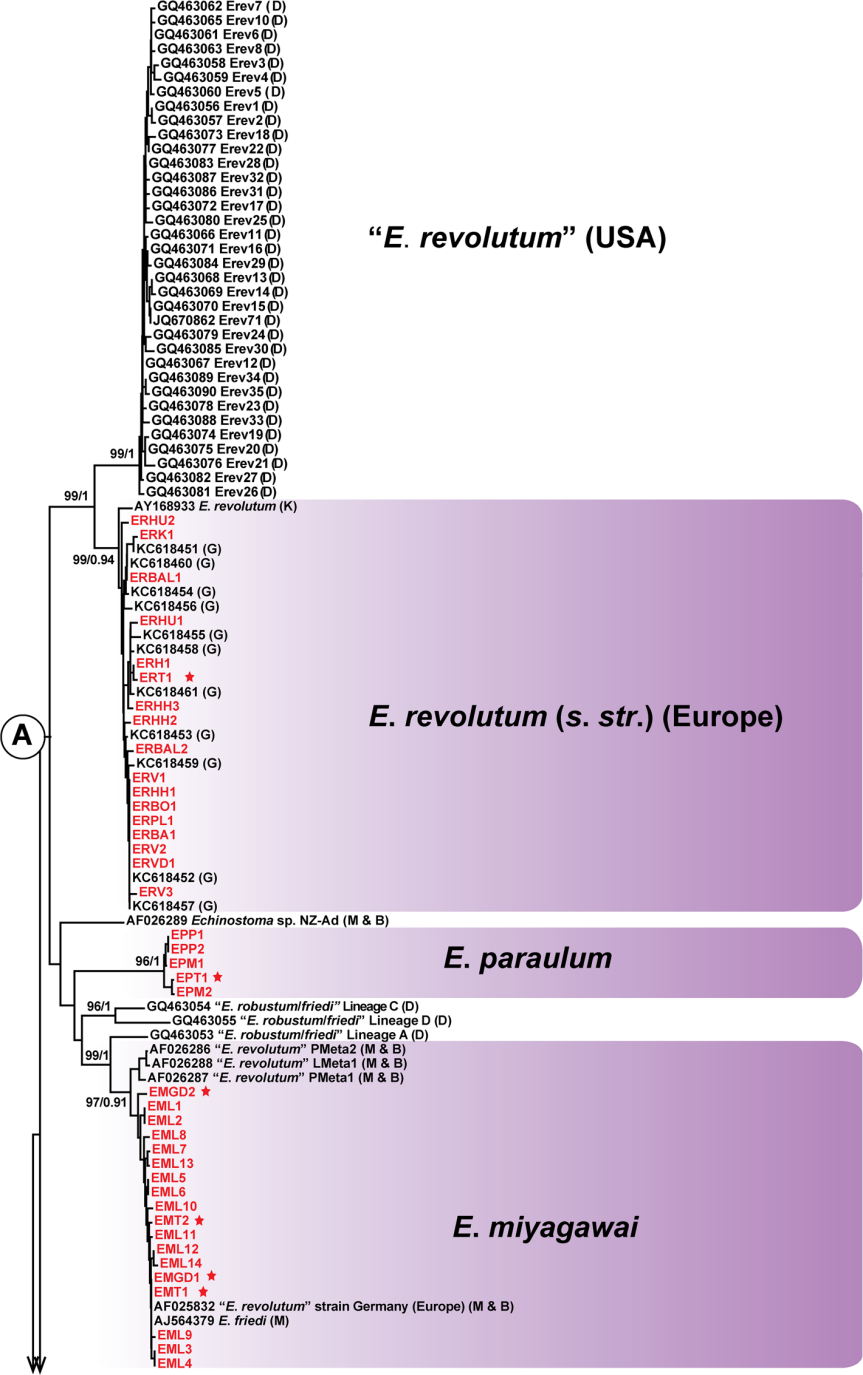
**Neighbour-Joining (NJ) tree for 16 species-level lineages within the '** ***revolutum*** **' group of** ***Echinostoma*** **based on the mitochondrial gene** ***nad*** **1: Clade A.** Based on a 475-nt fragment of *nad*1. Outgroups: *Echinoparyphium aconiatum* and *Hypoderaeum conoideum*. Numbers represent node supports from NJ and Bayesian inference (50% majority rule consensus tree) analyses (only values greater than 70 and 0.95, respectively, are shown). The newly-sequenced European isolates are shown in red; stars indicate adult isolates from natural infections. Sequence identification is as in GenBank, followed by a letter: D, Detwiler *et al*. [16,17]; G, Georgieva *et al*. [19]; K, Kostadinova *et al*. [28]; M, Marcilla *et al*. (unpublished); M & B, Morgan & Blair [14,26]. The scale-bar indicates the expected number of substitutions per site.

**Figure 2:**
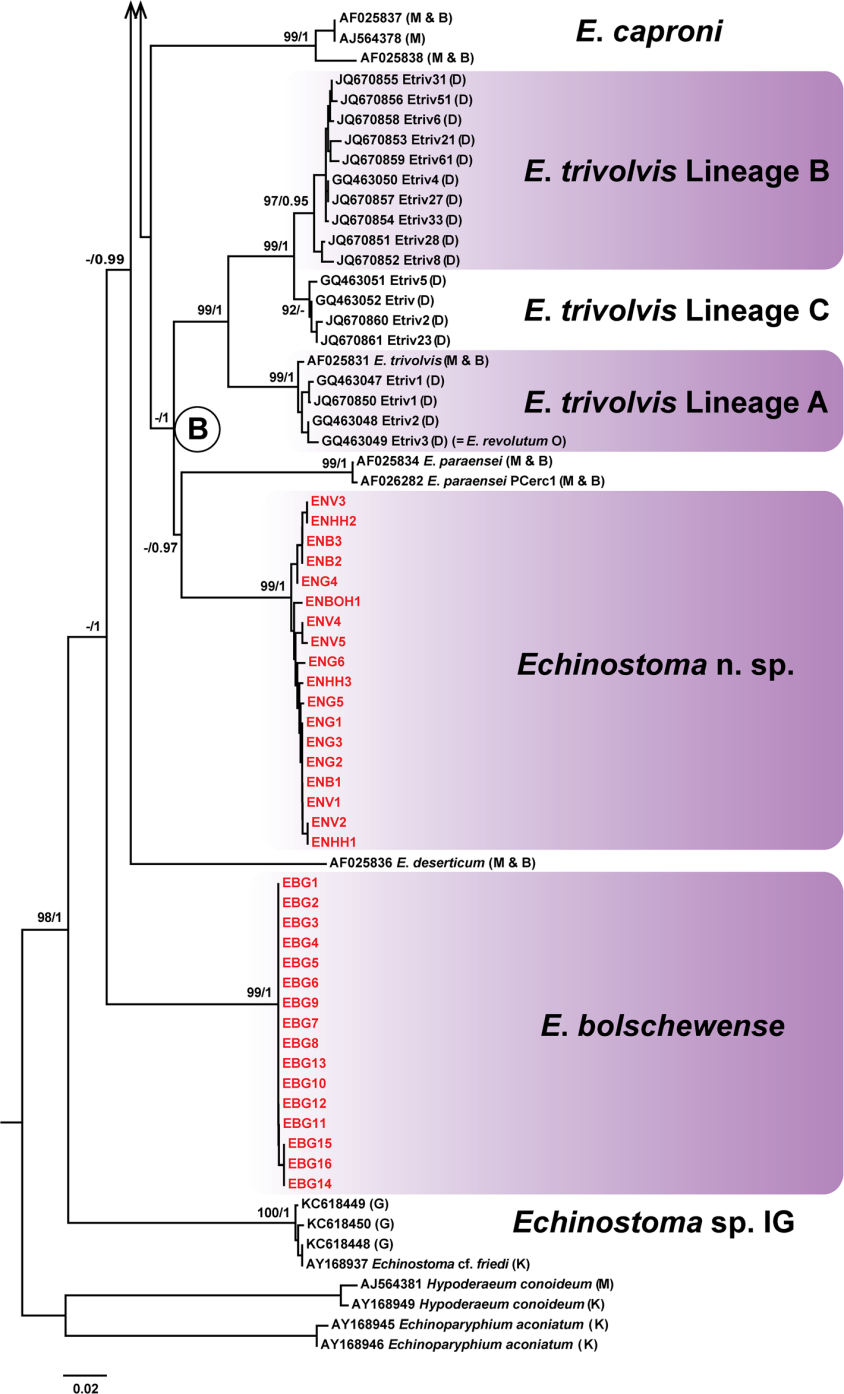
**Neighbour-Joining (NJ) tree for 16 species-level lineages within the '** ***revolutum*** **' group of** ***Echinostoma*** **based on the mitochondrial gene** ***nad*** **1: Clade B and the remaining species, continuation of Figure** 1 **.** The newly-sequenced European isolates are shown in red; stars indicate adult isolates from natural infections. Sequence identification is as in GenBank, followed by a letter: D, Detwiler *et al*. [16,17]; G, Georgieva *et al*. [19]; K, Kostadinova *et al*. [28]; M, Marcilla *et al*. (unpublished); M & B, Morgan & Blair [14,26]; O, Olson *et al*. [47]. The scale-bar indicates the expected number of substitutions per site.

Both NJ and BI analyses resulted in consensus trees with similar topologies. Figures 1 and 2 represent the hypothesis for the relationships within the ‘*revolutum*’ complex inferred from genetic distances (with indication of the nodal support from the BI analysis) of the *nad*1 dataset (159 sequences, 475 nt) that incorporated the sequences published by Morgan & Blair [14,26] (n = 11), Detwiler *et al*. [16,17] (n = 43), Georgieva *et al*. [19] (n = 14) and Kostadinova *et al*. [28] (n = 2); two otherwise unpublished sequences [AJ564379 (*E. friedi*) and AJ564378 (*E. caproni*)] of Marcilla *et al*. available on GenBank were also included in the analyses. NJ and BI analyses produced congruent results with minor topological differences. Six of the previously recognised species/cryptic lineages were represented by singletons thus preventing calculation of bootstrap support; however, most of these formed independent branches on the NJ and BI trees (Figures 1 and 2).

The newly-generated sequences from Europe fall into six distinct well-supported reciprocally monophyletic lineages corresponding to the species identifications based on morphology: *E. revolutum* ex *L. stagnalis*, *R. auricularia*, *R. peregra*, *S. palustris* and *Ay. fuligula*; *E. miyagawai* ex *P. planorbis*, *An. platyrhynchos* and *Ay. fuligula*; *E. paraulum* ex *L. stagnalis* and *Ay. fuligula*; *E. bolschewense* ex *V. acerosus*; *Echinostoma* sp. IG ex *R. auricularia* and *R. peregra*; and *Echinostoma* n. sp. ex *P. corneus*. Three species, *Echinostoma* sp. IG, *E. bolschewense* and *E. deserticum* (a laboratory strain from Niger maintained by Dr J. Jordane (France) with sequences previously reported as *Echinostoma* sp. I by Morgan & Blair [14,25,26]), appeared with maximum support as the earliest species to diverge among the ‘*revolutum*’ group. The remaining species/lineages formed two main clades (A and B), shown in Figures 1 and 2, respectively.

**Figure 3:**
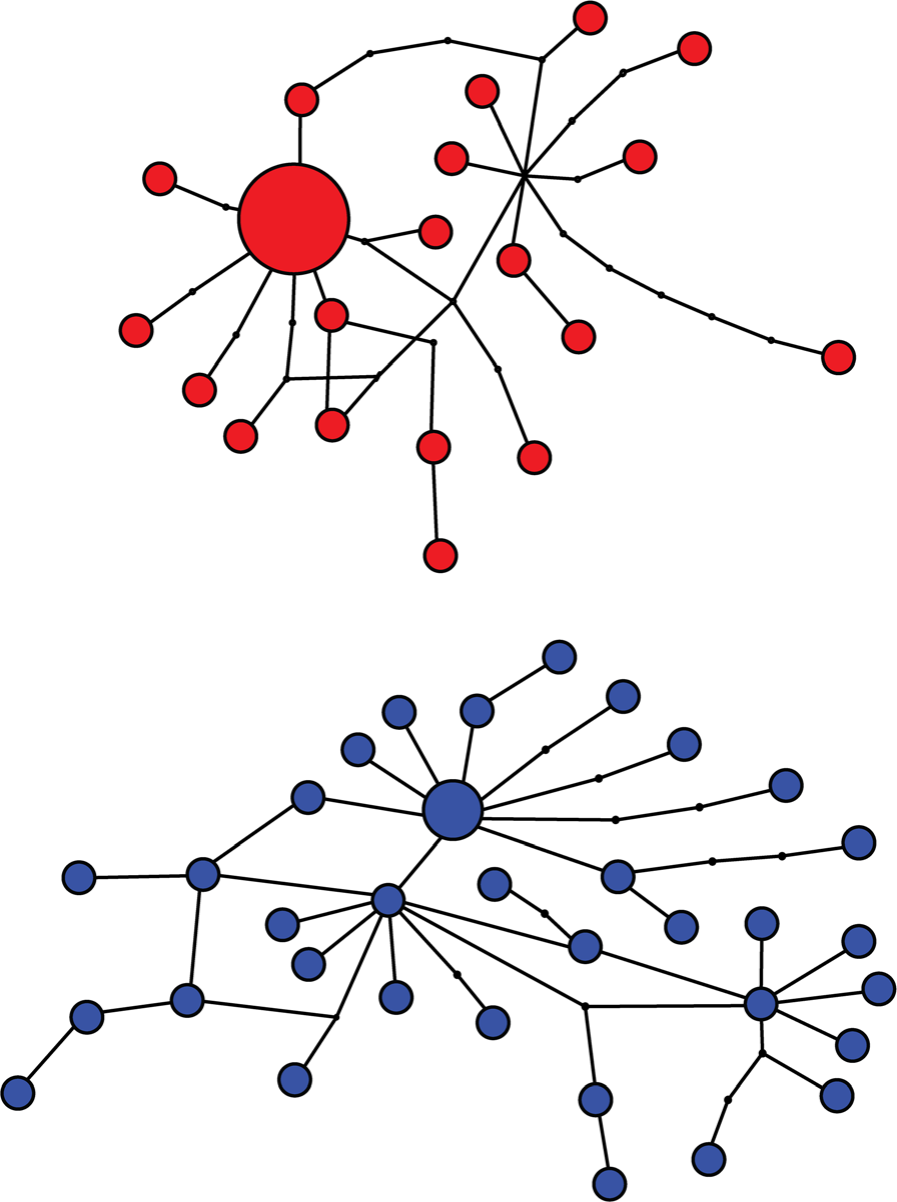
**Haplotype network for isolates of** ***Echinostoma revolutum*** **(** ***sensu lato*** **)** ***.*** Isolates of *E. revolutum* (*sensu stricto*) sampled in Europe (present study; Kostadinova *et al*. [28]; Georgieva *et al*. [19]) are shown in red and isolates of “*E. revolutum*” sampled in the USA by Detwiler *et al*. [16,17] are shown in blue.

The first clade (A) comprised the isolates of *E. revolutum sensu lato* (*s.l*.), *Echinostoma* sp. NZ-Ad, *E. paraulum*, *E. miyagawai* and the three lineages (labelled A–C) of “*E. robustum*/*friedi*” *sensu* Detwiler *et al*. [16,17] (Figure 1). Within this clade, the isolates ex *Stagnicola elodes* from the USA labelled as “*E. revolutum*” by Detwiler *et al*. [16,17] and the European isolates from four species of lymnaeids and wild and experimentally raised adults identified by us as *E. revolutum sensu stricto* (*s. str.*) based on morphology (see also [28]), formed sister reciprocally monophyletic lineages (Figure 1) with high support (as in Georgieva *et al*. [19]). The average sequence divergence between the two lineages was 5.9% and there were no shared haplotypes; the average intra-lineage divergence was low (0.88 and 0.83%, respectively; Table 2). Maximum parsimony haplotype network analysis depicted two unconnected networks at 95% connection limit for the isolates of *E. revolutum* (*s.l*.) from Europe and the USA (Figure 3). These results strongly support the suggestion of Georgieva *et al*. [19] that the North American isolates of “*E. revolutum*” of Detwiler *et al*. [16,17] represent a distinct cryptic species of the ‘*revolutum*’ group.

The European cercarial and adult isolates of *E. miyagawai* clustered together with: (i) one North American isolate (GQ463053), Lineage A of “*E. robustum*/*friedi*” *sensu* Detwiler *et al*. [16,17]; (ii) the isolate “*E. revolutum* Germany, Europe” (AF025832) of Morgan & Blair [14,25,26]; (iii) three Australian isolates (AF026286–AF026288) identified as *E. revolutum* by Morgan & Blair [14] and representing Lineage B of “*E. robustum*/*friedi*” *sensu* Detwiler *et al*. [16,17]; and (iv) the isolate of *E. friedi* of Marcilla *et al*. (AJ564379; sequence otherwise unpublished). The isolates (ii) and (iv) shared the most common haplotype of *E. miyagawai* from Europe thus confirming their conspecificity. When the North American isolate (i) was considered separately, the average intraspecific divergence for *E. miyagawai* was 0.83% and the average divergence between this isolate and *E. miyagawai* was 4.9% (range 4.2–5.3%) (Table 2). Surprisingly, the North American “*E. robustum*/*friedi*” of Detwiler *et al*. [16] was recovered as paraphyletic with lineages C and D divergent from Lineages A and B (i and iii above) (Figure 1) and comprising a pair of sister taxa that exhibited a strongly supported sister-group relationship with the European *E. paraulum* in the BI analysis.

The second clade (B) was characterised by maximum support at almost all nodes and comprised isolates of *Echinostoma* n. sp., *E. paraensei* and the isolates of the three lineages (A–C) of *E. trivolvis* identified by Detwiler *et al*. [16,17], joined by three isolates of *E. caproni* (NJ analysis only; Figure 2). There was poor support for Lineage C of *E. trivolvis* in the BI tree.

Overall, the analyses of the *nad*1 dataset provided evidence for 12 monophyletic groups and five singletons, which represent seven described/named species of *Echinostoma*, i.e. *E. revolutum* (*s. str*.), *E. bolschewense*, *E. caproni*, *E. deserticum*, *E. miyagawai*, *E. paraensei* and *E. paraulum*), and ten cryptic species-level lineages: *Echinostoma* n. sp. and *Echinostoma* sp. IG from Europe; “*E. revolutum*”, three lineages (A–C) of *E. trivolvis* (*s.l*.) and three lineages (A, C and D) of “*E. robustum*/*friedi*” *sensu* Detwiler *et al*. [16,17] from the USA; and *Echinostoma* sp. from New Zealand. Notably, the identification of the newly-sequenced adult isolates based on morphology alone, using the concept of Kostadinova *et al*. [1,2,28] for *E. revolutum* (*s. str*.), *E. miyagawai* and *E. paraulum*, matched the identification using molecular data.

**Figure 4:**
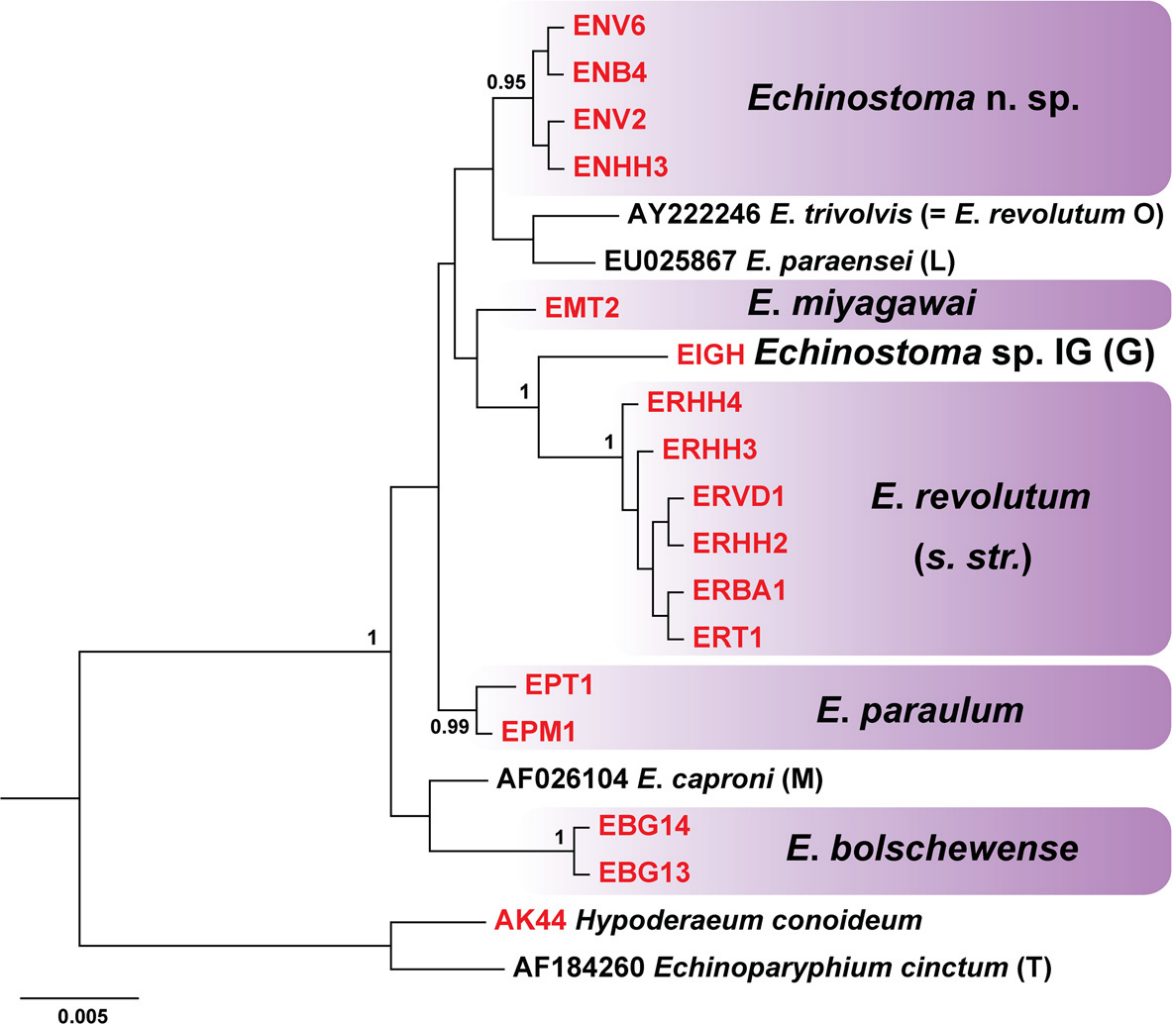
**Bayesian inference phylogram reconstructed using partial 28S rDNA sequences for nine** ***Echinostoma*** **spp.** The alignment comprised 1,219 nucleotide positions. Outgroups: *Hypoderaeum conoideum* and *Echinoparyphium cinctum*. The newly-sequenced European isolates are shown in red. Sequence identification is as in GenBank, followed by a letter: G, Georgieva *et al*. [19]; O, Olson *et al*. [47]; L, Lotfy *et al*. [49]; M, Mollaret *et al*. [50]. The scale-bar indicates the expected number of substitutions per site.

The 16 newly-generated 28S rDNA sequences corroborated with strong support the distinct species status of the six *nad*1 lineages of *Echinostoma* spp. studied in Europe (Figure 4). The only supported sister-group relationship was between *E. revolutum* and *Echinostoma* sp. IG but this is likely due to the incomplete taxon sampling for the 28S rRNA gene. No intraspecific variation was detected for species with multiple sequences, i.e. *E. revolutum*, *Echinostoma* n. sp. and *E. bolschewense*, and the two sequences (from one cercarial and one adult isolate) for *E. paraulum* differed at a single nucleotide position. The lower divergence range was 3–5 nucleotide positions (0.25–0.41%) between *Echinostoma* n. sp. and *E. paraulum*, *E. trivolvis*, *E. miyagawai* and *E. paraensei*; *E. paraulum* and *E. miyagawai*; and *E. paraensei* and *E. trivolvis* and *E. paraulum* (see Table 2 for details).

### Distance-based species delimitation

**Figure 5:**
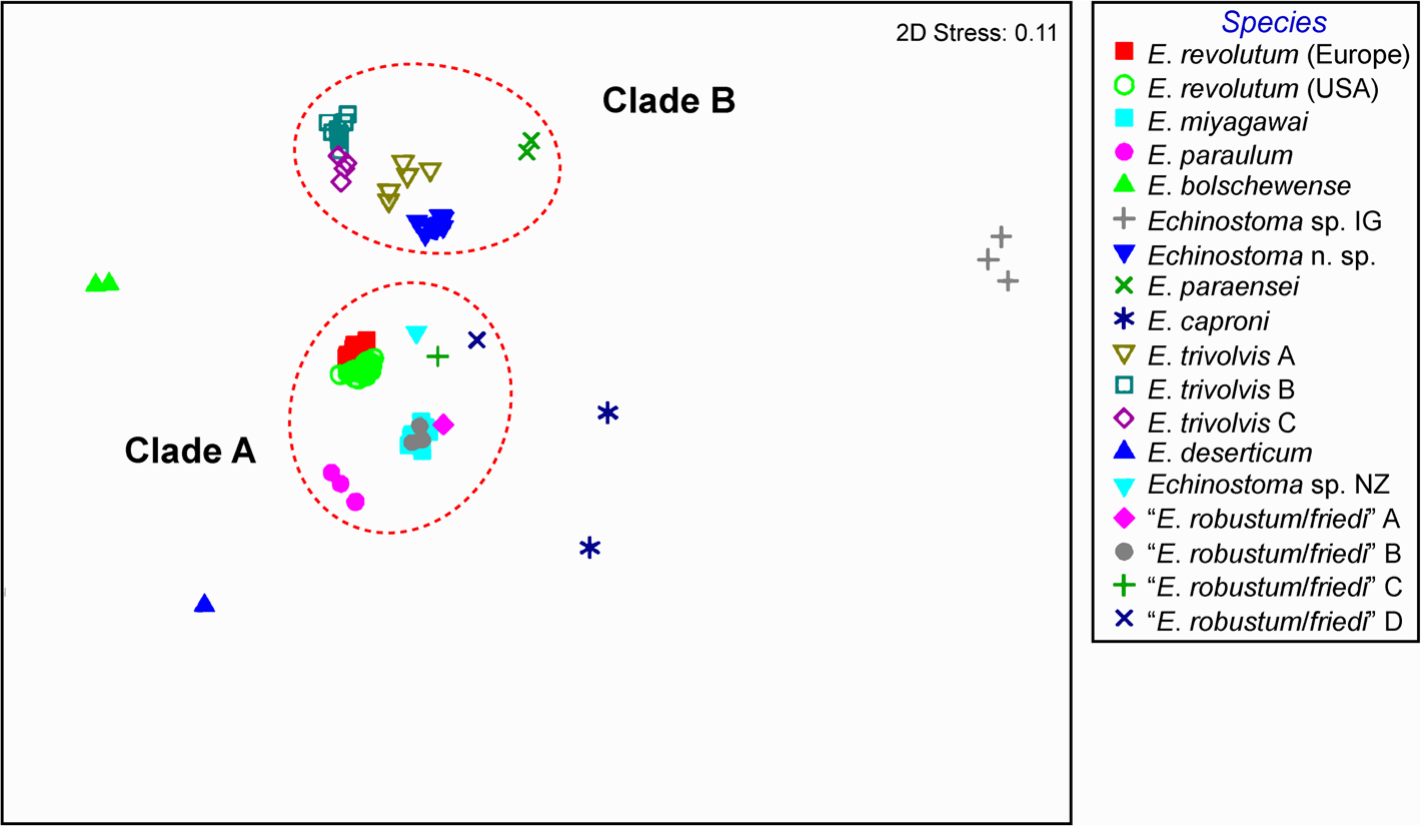
**Non-metric multidimensional scaling ordination plot derived from the raw pairwise distances calculated for the** ***nad*** **1 dataset.** Labels for the lineages of *E. trivolvis* and “*E. robustum/friedi*” are after Detwiler *et al*. [16]. Ellipses indicate the two main clades.

**Figure 6:**
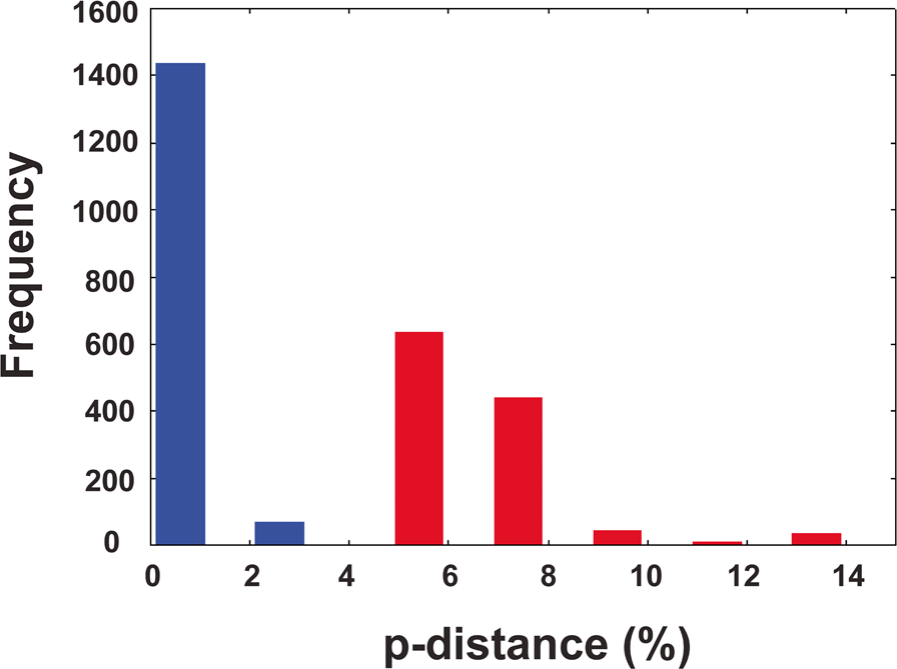
**Patterns of intra- and interspecific divergence in** ***Echinostoma*** **spp. using the** ***nad*** **1 dataset.** Red bars indicate intraspecific divergence; blue bars indicate iterspecific divergence (raw p-distances in %, pairwise comparisons between sister-species only).

The NMDS two-dimensional plot based on raw pairwise divergence data for all isolates with indication of the content of the two main clades discussed above is presented in Figure 5. The mean intraspecific divergence within the *nad*1 dataset was 0.81% (S.D. = 0.57%; range for mean divergence values of 0.21–1.82%; range for raw values of 0–3.59%, with just four comparisons exceeding 3%; see Table 2). These values were much lower than the mean divergence of 13.3% (S.D. = 3.1%) in the interspecific comparisons (range for mean divergence values of 2.7–19.6%; range for raw divergence values of 4.2–21.5%). There was no significant correlation between the number of isolates per species/lineage and mean intraspecific variation (Spearman’s rho = 0.248; *P* > 0.05). The mean interspecific divergence was 16-fold higher than mean intraspecific divergence but three sister-species groups [*E. trivolvis* Lineages A–C; *E. miyagawai* – “*E. robustum*/*friedi*” Lineage A; *E. revolutum* (*s. str*.) (Europe) – “*E. revolutum*” (USA)] exhibited ratios at the margin or below the ‘10× rule’ proposed by Hebert *et al*. [48], thus indicating a possible problem of overlapping variability at *nad*1 in the ‘*revolutum*’ species complex (see also Figure 5). However, there was no overlap in the distributions of intraspecific and interspecific (sister-taxa only) divergences (Figure 6). Furthermore, all sister-species groups could be resolved using diagnostic nucleotide sites: 65 for *Echinostoma* n. sp. – *E. paraensei*; 44 and 47 for *E. paraulum* – “*E. robustum*/*friedi*” Lineages C and D of Detwiler *et al*. [16], respectively; 28 for *E. trivolvis* Lineage A – *E. trivolvis* Lineages B and C; 24 for “*E. robustum*/*friedi*” Lineage C – “*E. robustum*/*friedi*” Lineage D of Detwiler *et al*. [16]; 19 for *E. revolutum* (*s. str*.) – “*E. revolutum*” (USA); and 16 for *E. miyagawai* – “*E. robustum*/*friedi*” Lineage A of Detwiler *et al*. [16]. Finally, excluding singletons, successful identification of all isolates was achieved for all 12 species/lineages at 3% divergence threshold in Species Identifier v.1.

## Discussion

The phylogenetic analyses depicted 17 genetically distinct lineages within the data set studied and, excluding singletons, successful identification of all isolates was achieved by the distance-based identification method implemented in Species Identifier v.1 for all 12 species/lineages. Our results are congruent with the phylogenies obtained by Detwiler *et al*. [16,17] on datasets dominated by isolates from the USA. The increase in the estimated number of species in the ‘*revolutum*’ group is largely due to the increased sampling within Europe. The novel sequence data generated here in association with the morphological characterisation of the life-cycle stages of *Echinostoma* spp. provides an integrative framework for future studies on species diversity within this difficult group.

### European species within the '*revolutum*' group

This first large-scale sequencing study of species of *Echinostoma* across Europe provided evidence for six molecularly distinct species of the ‘*revolutum*’ group. Their independent status was supported by the concordant signal of the mitochondrial *nad*1 and nuclear 28S rRNA genes, distance-based identification and morphological evidence. The integration of molecular and morphological data for two of the species-level lineages strongly indicates that these represent species new to science (see Georgieva *et al*. [19] for a description of the cercaria of *Echinostoma* sp. IG and Faltýnková *et al*. [46] for a description of the life-cycle stages of *Echinostoma* n. sp.).

Our extensive sampling resulted in a successful match of sequences based on life-cycle stages from naturally infected intermediate and definitive hosts for three of the European species whose life-cycles have been completed experimentally, *E. revolutum*, *E. miyagawai* and *E. paraulum* (see [1,2,46]). Notably, the identification of the adult isolates from natural infections based on morphology alone using the concept of Kostadinova *et al*. [1,2,28] and the morphological data from adult experimental isolates, matched the identification using molecular data. Sequencing of isolates from wild mammalian hosts within Europe may contribute to resolving the natural definitive hosts in the life-cycles of *E. bolschewense* and *Echinostoma* n. sp. The large-scale sampling of natural snail populations also shed light on the intermediate host range of *Echinostoma* spp. Whereas *E. bolschewense*, *E. miyagawai*, *E. paraulum* and *Echinostoma* n. sp. were found to infect single first intermediate snail species (*Viviparus acerosus*, *Planorbis planorbis*, *Lymnaea stagnalis* and *Planorbarius corneus*, respectively), *Echinostoma* sp. IG was detected in two snail hosts (*Radix auricularia* and *R. peregra*) and *E. revolutum* (*s. str.*) exhibited the widest intermediate host range (*L. stagnalis*, *R. auricularia*, *R. peregra* and *Stagnicola palustris*). These results further stress the importance of precise identification of cercarial isolates of *Echinostoma* spp. in hosts found to harbour more than one species: *L. stagnalis* (parasitised by two species, *E. revolutum* (*s. str*.) and *E. paraulum*), *R. auricularia* (*E. revolutum* (*s. str*.) and *Echinostoma* sp. IG) and *R. peregra* (*E. revolutum* (*s. str*.) and *Echinostoma* sp. IG). As shown by Georgieva *et al*. [19] and Faltýnková *et al*. [46], these species combinations can be distinguished based on cercarial morphology.

Perhaps the most important result of our study is that the integration of morphological and molecular data from both experimental and wildlife infections clarified the status of *E. revolutum* (*s. str*.) and *E. paraulum*. Both species use *L. stagnalis* as the first intermediate host but the cercariae differ in the number and location of the paraoesophageal gland-cells. The cercarial isolates from *L. stagnalis*, with a pattern of paraoesophageal gland-cells dissimilar to *E. revolutum* and experimentally obtained and wild adult isolates, formed a distinct strongly-supported clade with “*E. robustum/friedi*” Lineages C and D of Detwiler *et al*. [16,17] as nearest neighbours (Figure 1). A detailed examination of adult morphology (experimental set and the voucher specimen from natural infection used for sequencing; see [46]) confirmed their identification as *E. paraulum*, a species long considered a synonym of *E. revolutum* (see e.g. [4,5]). Combining morphological and molecular evidence from different life-cycle stages, we can confidently restore the validity of this species. All life-cycle stages of *E. revolutum* (*s. str.*) and *E. paraulum* linked to the sequences from Europe reported here are described in detail by Faltýnková *et al*. [46].

Our study provided the first datasets of sequences for *E. miyagawai* and *E. bolschewense. Echinostoma miyagawai* was re-validated after experimental completion of its life-cycle and detailed re-description of the morphology of all stages based on European material [1,2]; however, no sequences for this species were available. The incorporation of a large set of sequences for larval and adult *E. miyagawai* in our analyses solved the taxonomy of the German and Australian isolates identified as *E. revolutum* by Morgan & Blair [14,26]. Kostadinova *et al*. [28] examined a single voucher specimen (Australian isolate PMeta-2) of Morgan & Blair [14] and concluded that the morphology of this adult worm suggests an affiliation to *E. robustum*. However, they stated “… at present we prefer not to favour this specific identification for the ‘Australian-German’ clade of *Echinostoma* sp., pending a redescription of both larval and adult stages”. The inclusion of the sequences for four of the “*E. revolutum*” isolates of Morgan & Blair [14,26] within the well-supported clade of *E. miyagawai* (containing both cercarial and adult isolates identified using the concept of Kostadinova *et al*. [1,2]) suggests that these, in fact, belong to the latter species. The “German” isolate of “*E. revolutum*” (a laboratory strain identified by I. Kanev and sequenced by Morgan & Blair [14,25,26]) clearly represents a misidentification. As shown by Kostadinova *et al*. [1] based on re-examination of the voucher material, the re-description of *E. revolutum* by Kanev [5] was based on a mixture of material and likely represents a composite of at least two species of the ‘*revolutum*’ group. The position of *E. friedi* of Marcilla *et al*. (Valencia, Spain; AJ564379; published in GenBank only) within the *E. miyagawai* clade supports the inclusion of this species among the synonyms of *E. miyagawai*. Moreover, “*E. revolutum* Germany, Europe” of Morgan & Blair [14,26] (AF025832) and *E. friedi* (Valencia, Spain; AJ564379) represented a haplotype shared with adult isolates of *E. miyagawai* ex *An. platyrhynchos* from Poland and *Ay. fuligula* from the Czech Republic. The close association of *E. friedi* with the Australian isolates of Morgan & Blair [14,26] listed above was also confirmed in the recent study of Detwiler *et al*. [16] on a different set of taxa. However, a mislabelling of the sequence for *E. friedi* of Marcilla *et al.* (AJ564379) as the sequence for an isolate of Kostadinova *et al*. [28] provisionally identified as *E.* cf. *friedi* (AY168937) leaves a wrong impression that the latter isolate also represents *E. friedi* (see Georgieva *et al*. [19] for detailed discussion). As shown by Georgieva *et al*. [19] and the present study, the isolate of Kostadinova *et al*. [28] belongs to an as yet undescribed species of *Echinostoma* (*Echinostoma* sp. IG); this is strongly supported in the present analyses.

The life-cycle of *Echinostoma bolschewense* (possible synonym *E. jurini* (Skvortsov, 1924) of Kanev *et al*. [7]; for detailed comment on taxonomy see Faltýnková *et al*. [46]) has been elucidated by Našincová [21] who described in detail the life-cycle stages (rediae and cercariae from naturally infected prosobranch snails, *Viviparus contectus*, metacercariae from a range of prosobranch and pulmonate snails and adults from hamsters) of this species. To the best of our knowledge, this is the only species of *Echinostoma* developing in prosobranch snails; our study elucidated another first intermediate host, *Viviparus acerosus*.

In addition to the large *nad*1 dataset, we also generated 28S rDNA sequences for the six European species of the ‘*revolutum*’ group; these can be used in future phylogenetic studies at the supraspecific level. The minima for sequence divergence (0.25–0.41%) between *Echinostoma* spp. for which 28S rDNA data were available are comparable with the minima observed between closely related but distinct digenean species (e.g. 0.2–0.4% in the Cryptogonimidae, see Miller & Cribb [51,52].

### American species within the '*revolutum*' group

The taxonomy of the American species of *Echinostoma* belonging to the ‘*revolutum*’ group is in urgent need of revision. First, consistent with the recent study of Georgieva *et al*. [19], we found strong evidence for genetic differentiation between the North American and European populations within *E. revolutum* (*s.l*.) as evidenced by the phylogenetic reconstructions and distance-based identification. Therefore, the increased sampling within Europe reinforces the results of the network analysis of *E. revolutum* (*s.l.*) indicating lack of gene flow between Europe and North America [16].

Secondly, although the *nad*1 dataset was substantially expanded, the same lineages of *E. trivolvis* and “*E. robustum*/*friedi*” were recovered as identified by Detwiler *et al*. [16,17] suggesting that the lineages within *E. trivolvis* (A–C) and “*E. robustum/friedi*” (A, C and D) *sensu* Detwiler *et al*. [16] may represent distinct, closely-related cryptic species. However, this finding calls for further molecular and taxonomic scrutiny. In particular, comprehensive sampling in both North and South America is required to enlarge the sample size for the three lineages of “*E. robustum*/*friedi*” (note that this label is no more appropriate in view of the synonymy indicated above; we use it just for consistency in referring to the isolates of Detwiler *et al*. [16,17] currently represented by singletons). This would provide data for testing the monophyly of the lineages and alternative hypotheses for patterns of diversification associated with e.g. specificity to the snail host or geography. The strong support for different sister-group relationships of the three isolates of “*E. robustum*/*friedi*” further reinforce our suggestion; it is also worth noting that one of the isolates (Lineage D) originates from naturally infected *Biomphalaria glabrata* in South America (Brazil; see Detwiler *et al*. [16], whereas the other two (Lineages A and C) represent cercarial isolates ex *Lymnaea elodes* in the USA. It is also necessary to test if the structuring inferred from the *nad*1 sequences (Detwiler *et al*. [16,17]; this study) is reflected in divergences in the nuclear genes and consistent differences in morphology.

Although species boundaries are delimited, naming the American species would appear the most complicated task. Five nominal species assigned by different authors to the ‘*revolutum*’ group have been described in North America (USA), i.e. *Echinostoma armigerum*; *E. callawayense* Barker & Noll in Barker, 1915; *E. coalitum*; *E. trivolvis* and *Echinoparyphium contiguum* Barker & Barston in Barker, 1915 [6,53,54], and further eight species have been described in South America (Brazil), i.e. *E. barbosai*; *E. erraticum* Lutz, 1924; *E. luisreyi* Maldonado, Vieira & Lanfredi, 2003; *E. microrchis* Lutz, 1924; *E. neglectum* Lutz, 1924; *E. nephrocystis* Lutz, 1924; *E. rodriguesi* Hsu, Lie & Basch, 1968; *E. paraensei* Lie & Basch, 1967 [24,55-59]. In contrast to the opinions of Beaver [4] and Kanev *et al*. [6] regarding the synonymy of all North American species listed above with *E. trivolvis*, detailed studies on the morphology of some of the South American species have revealed that these exhibit distinguishing differences [18,24,57,59]. Comparative approaches to the morphology of North American strains of “*E. revolutum*” and *E. trivolvis* during the ‘pre-molecular era’ have shown that morphometric features of the experimentally raised adult worms can be used to distinguish closely related species [60,61].

Therefore, although the sequence information and analyses of Detwiler *et al*. [16,17] and the present study provide a sound framework for alpha taxonomy, revealing the species diversity of the ‘*revolutum*’ group of *Echinostoma* in the Americas requires an integrative approach linking the molecular data with detailed phenotypical characterisation of the isolates studied. Although the species within this group qualify as cryptic, the comprehensive morphological analysis in the course of our study revealed useful features for distinguishing two life-cycle stages, cercariae and adults, of the European *Echinostoma* spp. (Faltýnková *et al*. [46]; see also [19]). This stresses the importance of detailed morphological examination of live cercarial isolates prior to sequencing and the availability of voucher specimens identified by experts for the adult isolates sequenced (e.g. present study – see Faltýnková *et al.* [46]; Maldonado *et al*. [18]). The latter, even if unidentified at the time of DNA sequence publication, are of primary importance for accelerating further integrative taxonomy studies. Unfortunately, although a large number (32) of adult specimens of “*E. revolutum*”, *E. trivolvis* (Lineages A–C) and “*E. robustum/friedi*” (Lineage D) (see Additional file 1: Table S1) from natural infections or raised experimentally were sequenced by Detwiler *et al*. [16,17], these have not been submitted to a museum collection.

### Asian species within the '*revolutum*' group

Several notes of caution are required before considering the recent papers on “*Echinostoma*” spp. reported recently from Asian locations (Saijuntha *et al*. [62-64]; Noikong *et al*. [65]). First, the authors should be aware that annotations in GenBank solely reflect the identification (in most cases not supported by voucher material and/or morphological data) of the authors submitting the sequences. Whereas the identifications based on comparisons with original species descriptions may be correct, failure to follow the subsequent taxonomic/systematic changes may results in ‘discoveries’ such as “Interestingly, this study revealed that *E. revolutum* was more closely aligned with *E. recurvatum* than the other species of genus *Echinostoma* (e.g., *E. malayanum*), contradicting traditional morphological taxonomy.” (Saijuntha *et al*. [63]) and “Interestingly, this study revealed that two species of genus *Echinostoma*, i.e. *E. revolutum* and *E. malayanum* do not cluster as a monophyletic clade and/or sister taxa.” (Saijuntha *et al*. [62]). Just reading the subtitle for this species in the taxonomic revision of Kostadinova & Gibson [66], i.e. “*Artyfechinostomum malayanum* (Leiper, 1911) Railliet, 1925 [Syns *Echinostoma malayanum* Leiper, 1911; *Euparyphium malayanum* (Leiper, 1911) Leiper, 1915; *Echinoparyphium malayanum* (Leiper, 1911) Skrjabin & Shul’ts, 1929]” makes it clear that *E. malayanum* has been transferred to the genus *Artyfechinostomum* Lane, 1915 by Railliet nearly a century ago and that the only different generic placements of this species are those of Leiper (in *Euparyphium*) and Skrjabin & Shul’ts (in *Echinoparyphium*). Therefore, there is nothing “contradicting traditional morphological taxonomy” since the clustering pattern in Saijuntha *et al*. [62] simply reflects a distinction at the generic level which the authors failed to recognise because of lack of knowledge on the taxonomy of the group. Along this line, *Echinostoma hortense* Asada, 1926 has been transferred to the genus *Isthmiophora* as *I. hortensis* (Asada, 1926) in the revision of Kostadinova & Gibson [66]. The examination of the experimental material of *E. hortense* used for obtaining the sequence data of Morgan & Blair [14,25,26] confirmed its affiliation to *Isthmiophora* (see Kostadinova *et al*. [28]). However, this species is still referred to as *E. hortense* by Saijuntha *et al*. [62] and Noikong *et al*. [65].

A second problem in recent studies on Asian echinostomatids is the failure to understand/integrate existing knowledge (e.g. re-identifications of sequenced isolates based on morphological evidence, e.g. *Echinoparyphium ellisi* (AF026791, isolate PMeta3 of Morgan & Blair [14,26]) and *Echinoparyphium hydromyos* (AF026290, isolate Rat-Ad of Morgan & Blair [14]) re-identified by Kostadinova *et al*. [28] based on examination of the available voucher material, are still being referred to as “*Echinostoma* sp.” (see Noikong *et al*. [65]).

Thirdly, there are wrong interpretations of published work, e.g. “These results were relatively concordant to a previous report by Kostadinova *et al*., 2003, which confirmed that not all species within the genus *Echinostoma* represent a monophyletic group.” (Saijuntha *et al*. [62]). In fact, the opening sentence of the section “Molecular identification and relationships between *Echinostoma*, *Echinoparyphium*, *Hypoderaeum* and *Isthmiophora*” in Kostadinova *et al*. [28] states: “Considering **the initial identification (as given by Morgan & Blair, 1998a, b) and the names of the taxa as existing at present in the GenBank database** (our emphasis), *Echinostoma* is represented as a paraphyletic taxon with *Echinoparyphium recurvatum* (ITS and ND1 trees) and *Isthmiophora melis* (ND1 trees), *Echinoparyphium aconiatum* (ND1 trees) and *Hypoderaeum conoideum* (ND1 trees) nested within it.” [28]. Unfortunately, the findings of the study of Kostadinova *et al*. [28] were not understood by Saijuntha *et al*. [62].

Fourthly, the original papers should be consulted in order that the correct origin of the material sequenced is identified. For example, Saijuntha *et al*. [63] assumed that the sequence U58102 of Morgan & Blair [25] was of an “Australian isolate”. The provenance of this isolate is not annotated in GenBank but is clearly identified (i.e. Germany, Europe) in the original papers (see Table 1 in Morgan & Blair [25,26], respectively). The status of this isolate was discussed by Kostadinova *et al*. [28] who suggested a provisional identification as *Echinostoma* cf. *robustum* based on the additional molecular data. Failure to detect the origin of this isolate has resulted in a wrong conclusion, i.e. “Moreover, the phylogenetic relationships of *E. revolutum* presented in the present study suggested that genetic clustering is related to the geographical origin of the isolates, i.e., the American isolates closely aligned to the European isolate, whereas the Australian isolate closely aligned to Southeast Asian isolates.” (Saijuntha *et al*. [63]). In fact, the isolate of “*E. revolutum*” from Thailand exhibits close affinity to the European isolate studied by Morgan & Blair [14,26], which we have shown to represent *E. miyagawai* (see above). Finally, to our astonishment we found out that not a single sequence has been deposited in GenBank from the sequencing study in Thailand by Noikong *et al*. [65]. The lack of evidence for further comparative evaluation renders the findings reported by these authors useless.

Overall, these problems with the recent molecular studies based on Asian echinostomatids result in a rather bleak picture with regard to the identity of the isolates sequenced. It is likely that the papers by Saijuntha and colleagues deal with two species of the ‘*revolutum*’ group, one misidentified as *E. revolutum* and one misidentified as “*E. recurvatum* 43–50 collar spines” (*E. recurvatum* is a species with 45 collar spines), both exhibiting affinities with *E. miyagawai*. Whereas the identification of *Artyfechinostomum malayanum* (as *Echinostoma malayanum* in their papers) may be correct, that of “*Hypoderaeum conoideum* 41–45 collar spines” is likely wrong. Species of *Hypoderaeum* possess 43–82 collar spines [67] so that the minimum number of spines provided for the isolate (i.e. 41–45) is probably a miscount. Further, *H. conoideum* is characterised by the possession of 47–53 spines [68], i.e. above the range given by Saijuntha *et al*. [62]. Unfortunately, no data other than a short *cox*1 (250 nt) sequence are available to check their identification of “*H. conoideum*”. All these considerations indicate that further molecular work based on precise identification of the Asian isolates associated with the description and deposition of vouchers is required in order to make progress in elucidating the species diversity of the ‘*revolutum*’ group in Asia.

### *Nad*1 for a barcode?

The first assessment of the usefulness of the partial mitochondrial *nad*1 gene sequences for species identification and inferring the relationships within the ‘*revolutum*’ group was carried out in a comparative framework by Morgan & Blair [26]. Their findings suggested that *nad*1 is diverging significantly faster than the *cox*1 and ITS gene regions and thus appears to be the most informative region. These authors reported interspecific sequence divergence for *nad*1 within the ‘*revolutum*’ group of 12.3–30.8% [26] and 9.6–30.8% [14]. However, the very high upper limits of these ranges were due to inclusion in their comparisons of “*Echinostoma*” *hortense*, which was shown to belong to a different echinostomatid genus, *Isthmiophora* [66]. Detwiler *et al*. [16] reported a range of 1.2–5.4% and 8.1–12.4% for *nad*1 mean intra- and interspecific genetic divergence, respectively, for three sibling species groups of the ‘*revolutum*’ complex designated as “*E. revolutum*”, *E. trivolvis* (Lineages A–C) and “*E. robustum/friedi*” (Lineages A–D).

These values are generally comparable to the ranges obtained in our study (i.e. means of 0.2–1.8% and 2.7–19.4%, respectively), the mean pairwise divergence within the named and putative species in the present expanded dataset being much lower than the data reported by Detwiler *et al*. [16]. Although *nad*1 differentiation within species-level lineages was generally low compared with divergences between species with cases where the same haplotype was detected in remote geographical locations [*E. revolutum* (*s. str*.) and *E. miyagawai*], the overall mean interspecific divergence was 16-fold higher than the mean intraspecific divergence. The molecular divergences among three sister-species groups (i.e. *E. trivolvis* Lineages A–C; *E. miyagawai* – “*E. robustum*/*friedi*” Lineage A; *E. revolutum* (*s. str*.) (Europe) – “*E. revolutum*” (USA)) were relatively low (range for means 2.7–8.6%). However, a barcode gap (i.e. a discontinuity in levels of intraspecific compared with interspecific genetic divergence) was detected and all sister-species groups could be resolved using diagnostic nucleotide sites.

## Conclusion

Taking into account that a large comparative database of sequences exists, we conclude that *nad*1 should be the first choice for large-scale barcode-based identification of the species of the ‘*revolutum*’ group of *Echinostoma*. Our study provides a comprehensive reference library for precisely identified isolates of the European species and highlights the importance of an integrative approach for species identification linking molecular, morphological and biological data.

## Additional file

Additional file 1:
**Summary data for** ***nad*** **1 sequences of** ***Echinostoma*** **spp. retrieved from the GenBank.**

## References

[1] Kostadinova A, Gibson DI, Biserkov V, Chipev N (2000). Re-validation of *Echinostoma miyagawai* Ishii, 1932 (Digenea: Echinostomatidae) on the basis of experimental completion of its life-cycle. Syst Parasitol.

[2] Kostadinova A, Gibson DI, Biserkov V, Ivanova R (2000). A quantitative approach to the evaluation of the morphological variability of two echinostomes, *Echinostoma miyagawai* Ishii, 1932 and *E. revolutum* (Frölich, 1802) from Europe. Syst Parasitol.

[3] Kostadinova A, Gibson DI, Fried B, Graczyk TK (2000). The Systematics of the Echinostomes. Echinostomes as Experimental Models for Biological Research.

[4] Beaver PC (1937). Experimental studies on *Echinostoma revolutum* (Frölich) a fluke from birds and mammals. Ill Biol Monogr.

[5] Kanev I (1994). Life-cycle, delimitation and redescription of *Echinostoma revolutum* (Frölich, 1802) (Trematoda: Echinostomatidae). Syst Parasitol.

[6] Kanev I, Dimitrov V, Radev V, Fried B (1995). Redescription of *Echinostoma trivolvis* (Cort, 1914) with a discussion of its identity. Syst Parasitol.

[7] Kanev I, Fried B, Dimitrov V, Radev V (1995). Redescription of Echinostoma jurini (Skvortzov, 1924) with a discussion of its identity and characteristics. Ann Naturhist Mus Wien.

[8] Kosupko GA (1969). [The morphological peculiarities of *Echinostoma revolutum* and *E. miyagawai* cercariae.]. Trudy VIGIS.

[9] Kosupko GA (1971). New data on the bioecology and morphology of *Echinostoma revolutum* and *E. miyagawai* (Trematoda: Echinostomatidae). Byull VIGIS.

[10] Kosupko GA: **[Criteria of the species*****Echinostoma revolutum*****, demonstrated on experimental material.].** In *Sbornik rabot po gel’mintologii posvyashchen 90-letiyu so dnya rozhdeniya akademika K.I. Skryabina.* Moscow: ‘Kolos’; 1971:167–175. In Russian.

[11] Kosupko GA: *[Morphology and Biology of Echinostoma revolutum Frölich, 1802 and Echinostoma miyagawai Ishii, 1932 (Trematoda: Echinostomatidae) Studied on Experimental Material.]*, PhD Thesis. Moscow: VIGIS; 1972. In Russian.

[12] Našincová V (1986). Contribution to the distribution and the life history of *Echinostoma revolutum* in Central Europe. Věst Českoslov Společ Zool.

[13] Kostadinova A (1995). *Echinostoma echinatum* (Zeder, 1803) *sensu* Kanev (Digenea: Echinostomatidae): a note of caution. Syst Parasitol.

[14] Morgan JAT, Blair D (1998). Mitochondrial ND1 gene sequences used to identify echinostome isolates from Australia and New Zealand. Int J Parasitol.

[15] Sorensen RE, Kanev I, Fried B, Minchella DJ (1997). The occurrence and identification of *Echinostoma revolutum* from North American *Lymnaea elodes* snails. J Parasitol.

[16] Detwiler JT, Bos DH, Minchella DJ (2010). Revealing the secret lives of cryptic species: examining the phylogenetic relationships of echinostome parasites in North America. Mol Phylogenet Evol.

[17] Detwiler JT, Zajac AM, Minchella DJ, Belden LK (2012). Revealing cryptic parasite diversity in a definitive host: echinostomes in muskrats. J Parasitol.

[18] Maldonado A, Locker ES, Morgan JAT, Rey L, Lanfredi RM (2001). Description of the adult worms of a new Brazilian isolate of *Echinostoma paraensei* (Platyhelminthes: Digenea) from its natural vertebrate host *Nectomys squamipes* by light and scanning electron microscopy and molecular analysis. Parasitol Res.

[19] Georgieva S, Selbach C, Faltýnková A, Soldánová M, Sures B, Skírnisson K, Kostadinova A (2013). New cryptic species of the '*revolutum*' group of *Echinostoma* (Digenea: Echinostomatidae) revealed by molecular and morphological data. Parasit Vectors.

[20] Našincová V (1992). [Trematode developmental stages in Czech aquatic snails and life-cycles of selected species of the family Omphalometridae and Echinostomatidae].

[21] Našincová V (1991). The life cycle of *Echinostoma bolschewense* (Kotova, 1939) (Trematoda: Echinostomatidae). Folia Parasitol.

[22] Toledo R, Muñoz-Antolí C, Esteban JG (2000). The life-cycle of *Echinostoma friedi* n. sp. (Trematoda: Echinostomatidae) in Spain and a discussion on the relationships within the ‘*revolutum’* group based on cercarial chaetotaxy. Syst Parasitol.

[23] Kechemir N, Jourdane J, Mas-Coma S (2002). Life cycle of a new African echinostome species reproducing by parthenogenesis. J Nat Hist.

[24] Maldonado A, Vieira GO, Lanfredi RM (2003). *Echinostoma luisreyi* n. sp. (Platyhelminthes: Digenea) by light and scanning electron microscopy. J Parasitol.

[25] Morgan JAT, Blair D (1995). Nuclear rDNA ITS sequence variation in the trematode genus *Echinostoma*: an aid to establishing relationships within the 37-collar-spine group. Parasitology.

[26] Morgan JAT, Blair D (1998). Relative merits of nuclear ribosomal internal transcribed spacers and mitochondrial CO1 and ND1 genes for distinguishing among *Echinostoma* species (Trematoda). Parasitology.

[27] Sorensen RE, Curtis J, Minchella DJ (1998). Intraspecific variation in the rDNA ITS loci of 37-collar-spined echinostomes from North America: implications for sequence-based diagnoses and phylogenetics. J Parasitol.

[28] Kostadinova A, Herniou EA, Barrett J, Littlewood DTJ (2003). Phylogenetic relationships of *Echinostoma* Rudolphi, 1809 (Digenea: Echinostomatidae) and related genera re-assessed via DNA and morphological analyses. Syst Parasitol.

[29] Blouin MS (2002). Molecular prospecting for cryptic species of nematodes: mitochondrial DNA versus internal transcribed spacer. Int J Parasitol.

[30] Faltýnková A, Našincová V, Kablásková L (2007). Larval trematodes (Digenea) of the great pond snail, Lymnaea stagnalis (L.) (Gastropoda, Pulmonata), in Central Europe: a survey of species and key to their identification. Parasite.

[31] Faltýnková A, Našincová V, Kablásková L (2008). Larval trematodes (Digenea) of planorbid snails (Gastropoda: Pulmonata) in Central Europe: a survey of species and key to their identification. Syst Parasitol.

[32] Tkach V, Pawlowski J (1999). A new method of DNA extraction from the ethanol-fixed parasitic worms. Acta Parasitol.

[33] Lockyer AE, Olson PD, Littlewood DTJ (2003). Utility of complete large and small subunit rRNA genes in resolving the phylogeny of the Neodermata (Platyhelminthes): implications and a review of the cercomer theory. Biol J Linn Soc Lond.

[34] Bray RA, Waeschenbach A, Cribb TH, Weedall GD, Dyal P, Littlewood DTJ (2009). The phylogeny of the Lepocreadioidea (Platyhelminthes, Digenea) inferred from nuclear and mitochondrial genes: Implications for their systematics and evolution. Acta Parasitol.

[35] Tkach VV, Littlewood DTJ, Olson PD, Kinsella JM, Swiderski Z (2003). Molecular phylogenetic analysis of the Microphalloidea Ward, 1901 (Trematoda: Digenea). Syst Parasitol.

[36] Tamura K, Stecher G, Peterson D, Filipski A, Kumar S (2013). MEGA6: molecular evolutionary genetics analysis version 6.0. Mol Biol Evol.

[37] Telford MJ, Herniou EA, Russell RB, Littlewood DTJ (2000). Changes in mitochondrial genetic codes as phylogenetic characters: two examples from the flatworms. Proc Natl Acad Sci U S A.

[38] Ronquist F, Teslenko M, van der Mark P, Ayres DL, Darling A, Hohna S, Larget B, Liu L, Suchard MA, Huelsenbeck JP (2012). MrBayes 3.2: efficient Bayesian phylogenetic inference and model choice across a large model space. Syst Biol.

[39] Guindon S, Gascuel O (2003). A simple, fast and accurate method to estimate large phylogenies by maximum-likelihood. Syst Biol.

[40] Darriba D, Taboada GL, Doallo R, Posada D (2012). jModelTest 2: more models, new heuristics and parallel computing. Nat Methods.

[41] Rambaut A, Drummond AJ: *Tracer v1.4.*; 2007. Available from http://beast.bio.ed.ac.uk/Tracer.

[42] Huelsenbeck JP, Ronquist F, Nielsen R, Bollback JP (2001). Bayesian inference of phylogeny and its impact on evolutionary biology. Science.

[43] Clarke KR, Gorley RN (2006). PRIMER v6: User Manual/Tutorial.

[44] Meier RK, Shiyang G, Vaidya PKLN (2006). DNA barcoding and taxonomy in diptera: a tale of high intraspecific variability and low identification success. Syst Biol.

[45] Clement M, Posada D, Crandall KA (2000). TCS: a computer program to estimate gene genealogies. Mol Ecol.

[46] Faltýnková A, Georgieva S, Soldánová M, Kostadinova A (2015). A re-assessment of species diversity within the ‘revolutum’ group of *Echinostoma* Rudolphi, 1809 (Digenea: Echinostomatidae) in Europe. Syst Parasitol.

[47] Olson PD, Cribb TH, Tkach VV, Bray RA, Littlewood DTJ (2003). Phylogeny and classification of the Digenea (Platyhelminthes: Trematoda). Int J Parasitol.

[48] Hebert PDN, Stoeckle MY, Zelmak TS, Francis CM (2004). Identification of birds through DNA barcodes. PLoS Biol.

[49] Lotfy WM, Brant SV, DeJong RJ, Le TH, Demiaszkiewicz A, Rajapakse RP, Perera VB, Laursen JR, Loker ES (2008). Evolutionary origins, diversification, and biogeography of liver flukes (Digenea, Fasciolidae). Am J Trop Med Hyg.

[50] Mollaret I, Jamieson BG, Adlard RD, Hugall A, Lecointre G, Chombard C, Justine J-L (1997). Phylogenetic analysis of the Monogenea and their relationships with Digenea and Eucestoda inferred from 28S rDNA sequences. Mol Biochem Parasitol.

[51] Miller TL, Cribb TH (2007). Two new cryptogonimid genera (Digenea, Cryptogonimidae) from *Lutjanus bohar* (Perciformes, Lutjanidae): analyses of ribosomal DNA reveals wide geographic distribution and presence of cryptic species. Acta Parasitol.

[52] Miller TL, Cribb TH (2007). Coevolution of *Retrovarium* n. gen. (Digenea: Cryptogonimidae) in Lutjanidae and Haemulidae (Perciformes) in the Indo-West Pacific. Int J Parasitol.

[53] Barker F (1915). Parasites of the American muscrat (*Fiber zibethicus*). J Parasitol.

[54] Cort WW (1914). Larval trematodes from North American freshwater snails (Preliminary report). J Parasitol.

[55] Lutz A (1924). Estudos sobre a evoluacão dos endotrematodes brazileiros. Mem Inst Oswaldo Cruz.

[56] Lie KJ, Basch PF (1966). Life history of *Echinostoma barbosai* sp. n. (Trematoda: Echinostomatidae). J Parasitol.

[57] Lie KJ, Basch PF (1967). The life history of *Echinostoma paraensei* sp. n. (Trematoda: Echinostomatidae). J Parasitol.

[58] Hsu KC, Lie KJ, Basch PF (1968). The life history of *Echinostoma rodriguesi* sp. n. (Trematoda: Echinostomatidae). J Parasitol.

[59] Kohn A, Fernandes BMM (1975). Sobre as especies do genero *Echinostoma* Rudolphi, 1809 decritas por Adolpho Lutz em 1924. Mem Inst Oswaldo Cruz.

[60] Fried B, Mueller TJ, Frazer BA (1997). Observations on *Echinostoma revolutum* and *Echinostoma trivolvis* in single and concurrent infections in domestic chicks. Int J Parasitol.

[61] Humphries JE, Reddy A, Fried B (1997). Infectivity and growth of *Echinostosma revolutum* (Frölich, 1802) in the domestic chick. Int J Parasitol.

[62] Saijuntha W, Sithithaworn P, Duenngai K, Kiatsopit N, Andrews RH, Petney TN (2011). Genetic variation and relationships of four species of medically important echinostomes (Trematoda: Echinostomatidae) in South-East Asia. Infect Genet Evol.

[63] Saijuntha W, Tantrawatpan C, Sithithaworn P, Andrews RH, Petney TN (2011). Genetic characterization of *Echinostoma revolutum* and *Echinoparyphium recurvatum* (Trematoda: Echinostomatidae) in Thailand and phylogenetic relationships with other isolates inferred by ITS1 sequence. Parasitol Res.

[64] Saijuntha W, Tantrawatpan C, Sithithaworn P, Andrews RH, Petney TN (2011). Spatial and temporal genetic variation of *Echinostoma revolutum* (Trematoda: Echinostomatidae) from Thailand and the Lao PDR. Acta Trop.

[65] Noikong W, Wongsawad C, Chai J-Y, Saenphet S, Trudgett A (2014). Molecular analysis of echinostome metacercariae from their second intermediate host found in a localised geographic region reveals genetic heterogeneity and possible cryptic speciation. PLoS Negl Trop Dis.

[66] Kostadinova A, Gibson DI (2002). *Isthmiophora* Lühe, 1909 and *Euparyphium* Dietz, 1909 (Digenea: Echinostomatidae) re-defined, with comments on their nominal species. Syst Parasitol.

[67] Kostadinova A, Jones A, Bray RA, Gibson DI (2005). Family Echinostomatidae. Keys to the Trematoda.

[68] Skrjabin KI, Bashkirova EY (1956). Family echinostomatidae. Osnovy Trematodologii.

